# MCF-YOLO: Consistency-Guided Cross-Modal Attention for Small-Object RGB-IR Detection

**DOI:** 10.3390/s26123938

**Published:** 2026-06-21

**Authors:** Xiang Yang, Mengyue Yang, Xiaolan Xie

**Affiliations:** 1 College of Computer Science and Engineering, Guilin University of Technology, Guilin 541006, China; 1994022@glut.edu.cn; 2 Guangxi Key Laboratory of Embedded Technology and Intelligent System, Guilin University of Technology, Guilin 541004, China

**Keywords:** cross-modal attention mechanism, small object detection, modality consistency constraint, RGB–IR fusion object detection, multi-level feature fusion

## Abstract

In low-light, occluded, and cluttered environments, single-modality RGB detectors are prone to false positives and missed detections. While infrared (IR) imaging provides relatively stable target visibility under poor illumination, it lacks texture and color information and is susceptible to background thermal noise and imaging variations. To address these limitations, this paper proposes an RGB–IR object detection network, named MCF-YOLO, consisting of three core components. First, the Cross-Modal Hierarchical Fusion (CMHF) module performs stage-wise alignment and fusion on multi-scale features, jointly modeling RGB texture details and IR thermal responses to exploit the structural and semantic complementarity between the two modalities. Second, the Soft Attention Regularization based on Attention Prior (SAR-AP) module derives attention priors from IR features to impose soft constraints on cross-modal attention maps. This mechanism helps the network maintain attention on target-relevant regions, thereby suppressing attention drift caused by low-light noise and complex backgrounds. Third, the Small-Object-Sensitive Detection Head (SOS-Head) processes high-resolution features to strengthen the representation of small targets, improving detection capability in long-range and occluded scenarios. In evaluations on two RGB–IR benchmarks—M3FD and VEDAI—MCF-YOLO achieves improvements of 2.7% in mAP@0.5 and 1.1% in mAP@0.5:0.95 on M3FD, and 5.4% and 4.4%, respectively, on VEDAI. These results suggest that consistency-guided cross-modal fusion and high-resolution small-target modeling are beneficial for RGB–IR detection in low-visibility and cluttered scenes.

## 1. Introduction

In autonomous driving and advanced driver-assistance systems (ADAS), reliable environmental perception is safety-critical for detecting pedestrians, vehicles, and other obstacles in complex scenarios. However, under low-visibility conditions—such as nighttime, dense fog, and severe backlighting—RGB imaging is vulnerable to illumination changes, sensor noise, and glare. These factors weaken or obscure target textures, leading to degraded detection performance. To mitigate this issue, recent research has increasingly integrated infrared (IR) sensors with visible-light cameras to build multispectral sensing systems [1]. Relying on thermal signatures rather than visible light, IR imaging can provide relatively stable target responses under poor illumination. Consequently, RGB–IR detection is generally more reliable than RGB-only detection in low-visibility scenes [2].

A key issue in multispectral object detection is how to fuse complementary cues from different modalities. Early studies often employed simple feature-level fusion operations, such as feature concatenation or element-wise addition. However, such operations are limited in handling cross-modal distribution discrepancies and feature misalignment, especially under complex backgrounds and modality interference [3]. Recent studies have moved toward cross-modal feature reconstruction and attention-guided fusion to strengthen modality interaction in multispectral detection [4].

Recent work has further investigated multi-level fusion and modality reweighting mechanisms to improve RGB–IR feature integration [5]. Despite these advances, existing fusion modules may still produce unstable responses or modality interference in degraded or cluttered scenes [6]. In addition, the thermal priors carried by IR features remain insufficiently exploited to guide cross-modal attention toward target-relevant regions.

Furthermore, detecting small objects in nighttime or foggy scenes remains challenging because these targets occupy few pixels and often exhibit low visual contrast. Existing studies have attempted to strengthen fine-grained structural representation for small objects using multi-scale modeling or enhanced context representation [7,8]. However, most small-target-oriented methods are designed for single-modality detection and do not fully exploit cross-modal fusion. Meanwhile, although recent multispectral frameworks have made progress [9], explicit multi-scale feature enhancement specifically tailored for small objects remains underexplored, which may lead to missed detections in long-distance scenarios.

To address these challenges, this paper proposes MCF-YOLO (Multi-level Cross-Modality Fusion YOLO), a single-stage RGB–IR detection architecture for complex autonomous driving scenarios. Built upon the YOLO11 framework, the main contributions of this paper are as follows:

1. A Cross-Modal Hierarchical Fusion (CMHF) module is proposed to perform stage-wise alignment and fusion of multi-scale RGB and IR features. By jointly modeling the complementarity between RGB textures and IR thermal responses, CMHF alleviates modality interference and strengthens cross-modal feature representation.
2. A Soft Attention Regularization module based on Attention Prior (SAR-AP) is introduced. By imposing soft constraints derived from IR features, SAR-AP encourages the network to maintain attention on target-relevant regions, thereby improving the stability of cross-modal feature fusion under low-light and cluttered conditions.
3. A Small-Object-Sensitive Detection Head (SOS-Head) is designed to strengthen the representation of small targets in high-resolution branches, improving the detection accuracy of small objects in long-distance and occluded scenarios.

Experiments on the M3FD multispectral dataset show that MCF-YOLO achieves 82.6% mAP@0.5 and 54.9% mAP@0.5:0.95. Furthermore, when trained from scratch on the VEDAI dataset, the proposed method achieves 72.9% mAP@0.5 and 45.0% mAP@0.5:0.95 on aerial imagery. To evaluate cross-dataset generalization, we conduct validation on the LLVIP dataset using weights trained on M3FD, achieving 44.0% mAP@0.5 and 22.3% mAP@0.5:0.95. These results indicate that MCF-YOLO maintains competitive performance across different RGB–IR detection settings, including multispectral road scenes, aerial imagery, and low-light pedestrian scenarios.

## 2. Related Work

### 2.1. Overview of Multispectral Fusion Object Detection Methods

#### 2.1.1. Motivation of Single-Modal Object Detection and Multispectral Fusion

One-stage detectors, particularly the YOLO series [10], have been widely used in real-time object detection because they offer a practical balance between accuracy and inference efficiency. YOLO11 [11] incorporates architectural refinements for feature extraction and provides a suitable baseline for the proposed multispectral detection framework.

However, RGB-only perception is inherently constrained by the imaging properties of visible light. In scenes with low illumination, strong glare, or smoke occlusion, RGB images often suffer from contrast degradation and texture loss, which weakens target discrimination. These limitations motivate the introduction of infrared (IR) information, as thermal cues can complement degraded visible textures under challenging illumination conditions.

#### 2.1.2. Objective of RGB–IR Feature Fusion

RGB–IR fusion aims to obtain more reliable target representations by exploiting the complementary properties of visible and infrared modalities. Visible images provide fine-grained color and texture details, whereas infrared images capture thermal responses that help preserve target contours under nighttime, backlit, or visually degraded scenarios. An effective fusion strategy should integrate structural and semantic cues from both modalities while reducing redundant or conflicting responses [12]. Formally, this fusion process can be generalized as

(1) $$F_{\mathrm{fused}}=\Phi \left(F_{\mathrm{RGB}},F_{\mathrm{IR}}\right)$$

where $F_{\mathrm{RGB}}$ and $F_{\mathrm{IR}}$ denote the feature tensors of the respective modalities, and Φ(·) represents the cross-modal integration function. Designing an adaptive Φ that balances modal contributions while suppressing sensor-specific noise remains a key challenge in multispectral research [13].

#### 2.1.3. Evolution of Fusion Hierarchies

A key design issue in multispectral detection is where cross-modal interaction should be introduced in the network. According to the fusion stage, existing methods can be broadly grouped into early fusion, mid/halfway fusion, and late/score fusion [14].

Early Fusion: Early fusion directly concatenates RGB and IR channels at the input or shallow feature layers. Although this strategy is simple to implement, it may cause premature feature entanglement and make it difficult to preserve the modality-specific characteristics of visible and infrared data.

Mid Fusion: Mid fusion performs cross-modal interaction within the backbone or neck stages. At this level, features retain certain structural details while gradually acquiring semantic abstraction, which often benefits multispectral representation. However, conventional mid-fusion designs usually rely on limited fusion stages and may not fully coordinate multi-scale modality information.

Late/Score Fusion: Late or score fusion combines modality-specific predictions at the detection head or through confidence-weighted ensembles. Such methods are flexible, but their interaction is mainly performed at the decision level and therefore provides limited deep feature complementarity.

Compared with early fusion or decision-level fusion, mid-level interaction is often regarded as a practical compromise between feature complementarity and architectural complexity.

#### 2.1.4. Fusion Trends and Challenges

Recent multispectral fusion methods have gradually moved beyond single-stage feature aggregation toward more adaptive and structured interaction designs. Existing studies can be broadly discussed from three perspectives: multi-level fusion organization, adaptive modality interaction, and alignment- or attention-guided fusion refinement. GLFusion explores global-local feature interaction to enhance multispectral representation across different receptive fields [15]. Guo et al. further investigate adaptive cross-modal interaction, aiming to dynamically model modality complementarity rather than simply aggregating heterogeneous features [16]. DACFusion performs asymmetric cross-attention fusion by considering the different characteristics of visible and infrared modalities [17]. EI2Det combines cross-modal interaction, illumination-aware weighting, and edge-guided fusion to adapt modality contributions under varying illumination conditions [18]. COXNet introduces cross-layer fusion with adaptive alignment and scale integration for RGB–IR tiny object detection [19]. These studies indicate that effective RGB–IR fusion requires not only adaptive modality interaction, but also coordinated feature exchange across different network stages.

In addition, several methods attempt to improve the reliability of fused features under modality misalignment, background interference, or unstable spatial responses. AR-CNN introduces a Region Feature Alignment (RFA) module to alleviate position shifts between weakly aligned visible and thermal image pairs [20]. DeformCAT further addresses weakly aligned RGB-T pedestrian detection by modeling deformable cross-modal spatial correlations through cross-attention [21]. Beyond alignment-oriented designs, EAEF enhances RGB-thermal fusion by explicitly modeling modality-wise attention responses for different perception tasks [22]. T-aware uses thermal images to guide early fusion and enhance salient regions under cross-illumination conditions [23]. TFDet adopts a target-aware fusion-refinement strategy to suppress background-related responses and reduce false positives caused by noisy fused features [24].

Although these methods improve RGB–IR fusion from different perspectives, most of them emphasize a specific fusion cue, such as global-local interaction, adaptive cross-modal interaction, cross-attention, illumination-aware weighting, edge guidance, cross-layer alignment, geometric alignment, thermal guidance, or target-aware refinement. In contrast, the proposed CMHF emphasizes a progressive organization of RGB-IR interaction across shallow, intermediate, and high-level feature stages, aiming to coordinate complementary modality cues in a hierarchical manner. Meanwhile, SAR-AP introduces an infrared-guided soft attention prior to regularize the spatial response of fused features. Different from hard geometric alignment or target-aware feature refinement, the “structural consistency” in this work refers to the stability and target-region coherence of fused attention responses, rather than the positional alignment between RGB and IR features.

### 2.2. Small-Object Detection and Multi-Scale Feature Enhancement Methods

In applications such as UAV monitoring, nighttime driving assistance, and long-range surveillance, small objects usually occupy only a limited number of pixels and are easily affected by background clutter, motion blur, and occlusion. During feature extraction in deep object detection networks, repeated down-sampling operations further weaken the spatial details of small instances. As a result, detectors may still suffer from missed detections and inaccurate localization even when high-resolution inputs are used.

Feature pyramid structures are commonly used to improve the representation of objects with large-scale variations. FPN and PAN construct top-down and bottom-up information flows to combine semantic and localization cues across different feature levels. For small objects, another direct strategy is to introduce high-resolution detection layers, such as extending the conventional P3–P5 pyramid with a P2 branch, so that more spatial and edge details can be retained for fine-grained localization.

Several YOLO-based small-object detection methods are closely related to this design. AGLC-YOLO introduces global-local context modeling and an additional small-object prediction head for UAV images [25]. PCPE-YOLO adds a small-object detection layer and enhances context-aware feature extraction for small targets [26]. YOLO-SSP improves remote-sensing small-object detection through an improved downsampling strategy, a small-object detection layer, and pyramid spatial attention [27]. ES-YOLOv8 constructs a refined multi-scale detection framework for detecting small floating objects in complex water-surface scenes [28]. Different from these single-modal small-object enhancement methods, SOS-Head is embedded in the RGB–IR fusion pipeline and operates on fused multispectral features, allowing high-resolution spatial details and cross-modal complementary cues to jointly contribute to small-object localization.

## 3. Methodology

### 3.1. Overall Architecture

The YOLO family provides a widely used single-stage detection framework with a favorable trade-off between detection accuracy and inference cost. Its backbone and feature-pyramid designs make it suitable for efficient multi-scale object detection.

While earlier versions such as YOLOv8n maintain competitive detection accuracy, YOLO11 has further refined the backbone and feature-pyramid components, improving feature extraction while reducing computational footprint. This efficiency leaves additional room for the integration of multispectral fusion modules without incurring excessive processing overhead. Consequently, we select YOLO11n as the baseline for MCF-YOLO, keeping the additional computational cost from dual-branch feature extraction and cross-modal interaction manageable.

**Figure 1:**
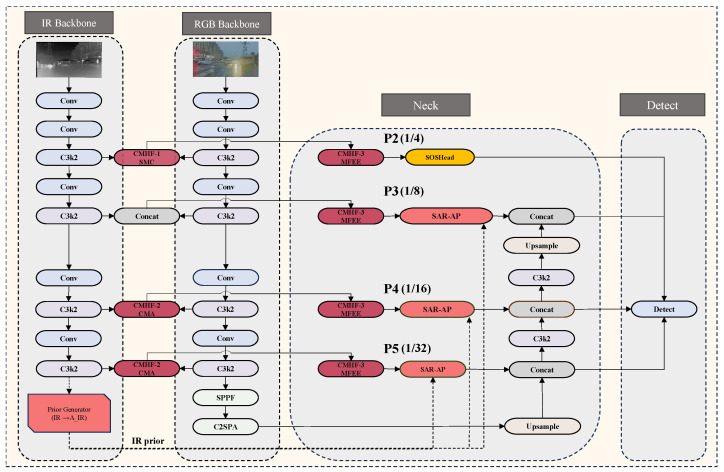
Overall architecture of MCF-YOLO.

The proposed MCF-YOLO is designed for RGB–IR multispectral scenarios. Accordingly, we adopt a design principle that maintaining decoupled representation spaces and utilizing multi-scale interaction mechanisms are more effective for exploiting modality complementarity than simple input concatenation. Structurally, MCF-YOLO is built upon four design principles: modality-decoupled representation, hierarchical cross-modal fusion, prior-guided consistency regularization, and small-object enhancement, as illustrated in Figure 1. The specific implementations of this architecture and its individual modules are detailed in the subsequent subsections.

Specifically, MCF-YOLO adopts a dual-branch backbone for RGB–IR feature extraction and incorporates the following three core modules:

1. Cross-Modal Hierarchical Fusion (CMHF): Operating on the dual-branch outputs, the CMHF module progressively integrates information across semantic levels. Shallow Modality Calibration (SMC) is applied at the high-resolution P2 layer, while Cross-Modal Attention (CMA) models bidirectional complementarity at the deeper P4–P5 layers. A Multi-Scale Fusion Feature Enhancement (MFEE) module then refines these fused features to produce stable representations.
2. Soft Attention Regularization with Attention Prior (SAR-AP): Integrated at the P3–P5 scales, the SAR-AP module introduces an infrared thermal prior to guide the fusion process. By imposing soft attention regularization based on the IR thermal map, this module improves response-level structural consistency and stability in regions with dense small objects or background interference.
3. Small-Object-Sensitive Detection Head (SOS-Head): To address the limitations of standard detection heads, a dedicated small-object branch is designed using P2 high-resolution features. This branch fuses with the mid-level semantic features of P3 and utilizes a specialized convolutional design to enhance the representation of small targets.

Together, CMHF, SAR-AP, and SOS-Head operate at different stages of the detection pipeline, respectively addressing hierarchical RGB–IR fusion, prior-guided response regularization, and small-object prediction.

### 3.2. Dual-Branch Backbone for RGB–IR Feature Extraction

In multispectral object detection, RGB and infrared (IR) modalities differ in imaging mechanisms, spectral responses, and texture characteristics. Directly concatenating the two modalities along the channel dimension and feeding them into a single-stream backbone may introduce cross-modal interference, resulting in unstable feature distributions and weakened discriminative representations [29]. To mitigate this issue, we adopt a dual-branch backbone based on YOLO11n, where RGB and IR inputs are processed by two separate branches with non-shared parameters. This design allows each branch to learn modality-specific representations in the early stages and reduces the potential suppression of weak thermal cues by dominant visible textures. It also follows the mid-fusion paradigm commonly used in multispectral object detection.

The RGB branch mainly extracts appearance-related cues, including texture details, color gradients, and fine spatial structures. In contrast, the IR branch captures thermal responses and structural contours, which are less sensitive to illumination degradation and can compensate for the information loss in the RGB stream under low-visibility conditions. Both branches inherit the hierarchical backbone design of YOLO11n and use the same basic building blocks, thereby preserving comparable network depth and receptive-field scales with the original topology. With independent convolutional parameters, the dual-branch structure decouples the modality-specific feature spaces before cross-modal interaction.

The dual-branch backbone outputs scale-corresponding feature maps at four stages, denoted as $\left\{P2_{i},P3_{i},P4_{i},P5_{i}\right\}$, where i∈{RGB,IR}. Here, “scale-corresponding” indicates that the RGB and IR features are extracted at the same resolution levels, rather than being explicitly spatially aligned. Compared with a single-stream structure, this dual-path design preserves modality-specific representations while remaining compatible with the standard YOLO11n framework. These multi-scale features provide the basis for the subsequent hierarchical fusion and attention regularization modules.

### 3.3. Cross-Modal Hierarchical Fusion (CMHF)

The hierarchical architecture of CMHF is motivated by the distinct feature representations across different network depths. Specifically, the shallow layer (P2) preserves high-resolution spatial details, making it suitable for local spatial calibration and channel-wise recalibration to alleviate initial modality discrepancies. In contrast, the deeper layers (P4–P5) contain highly abstracted semantic information, where cross-modal attention is used to exploit global semantic complementarity and long-range dependencies between RGB and IR modalities. This progressive strategy allows shallow calibration to provide a more stable feature basis for mid-to-high-level semantic interaction.

Effectively fusing RGB and infrared features remains a key challenge in multispectral detection. Some existing methods directly concatenate RGB and IR features at the input or shallow stages and use adaptive gating to alleviate information conflicts [30]. In this paper, we propose the CMHF module within the YOLO11 framework, which decomposes the fusion process into three specialized stages: Shallow Modality Calibration (SMC), Cross-Modal Attention (CMA), and Multi-Scale Fusion Feature Enhancement (MFEE).

SMC performs channel-wise recalibration and local spatial refinement to alleviate modality inconsistencies in the early stages. Subsequently, CMA is introduced at the mid-to-high layers to explicitly model bidirectional semantic interaction. Finally, MFEE is applied to enhance and re-weight the multi-scale fused features, suppressing redundant background noise while highlighting task-relevant target regions. Furthermore, the multi-scale features generated by CMHF serve as important inputs for the Small-Object-Sensitive Detection Head (SOS-Head), helping the network maintain high sensitivity to fine-grained details in complex environments.

#### 3.3.1. Shallow Modality Calibration (SMC)

Standard early-fusion strategies that directly concatenate RGB and IR features can introduce inter-modality interference, amplifying statistical distribution discrepancies and energy imbalances in the shallow layers. To mitigate these inconsistencies, we introduce the Shallow Modality Calibration (SMC) module. Before mid-level interaction, SMC performs explicit statistical (channel-wise) and geometric (spatial) calibration on the RGB and IR streams independently, reducing the representational burden on subsequent fusion stages.

**Figure 2:**
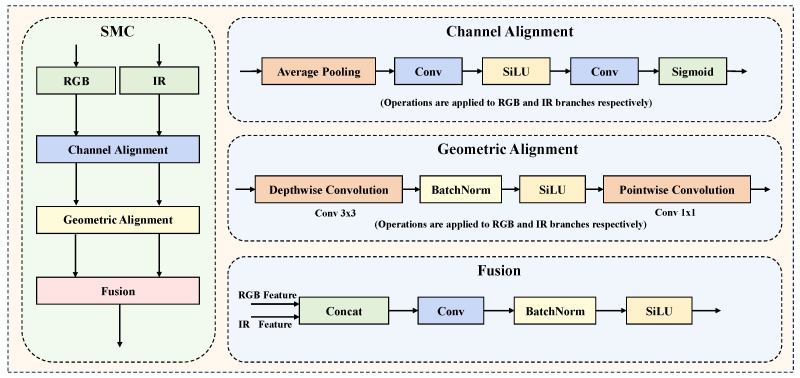
SMC structure diagram.

Given an input resolution of 640×640, the multi-scale features are downsampled hierarchically. Let the RGB and IR features output by the backbone at the P2 layer (with a downsampling factor of 4) be

(2) $$F_{RGB}^{\left(2\right)},F_{IR}^{\left(2\right)}\in R^{C_{2}\times H_{2}\times W_{2}}$$

where $H_{2}=W_{2}=160$, and $C_{2}$ denotes the channel dimension. To suppress redundant channels and emphasize informative semantic cues, SMC applies channel-wise recalibration to each modality [31]. As illustrated in Figure 2, global features are first extracted via Global Average Pooling (GAP), followed by a multi-layer perceptron (MLP) consisting of two 1×1 convolutions and a SiLU activation to generate weight vectors $s_{RGB}$ and $s_{IR}$:

(3) $$s_{RGB}=\sigma \left(\mathrm{MLP}\left(\mathrm{GAP}\left(F_{RGB}^{\left(2\right)}\right)\right)\right),\;s_{IR}=\sigma \left(\mathrm{MLP}\left(\mathrm{GAP}\left(F_{IR}^{\left(2\right)}\right)\right)\right)$$

where σ(·) is the Sigmoid activation function. The recalibrated features are obtained via element-wise multiplication ⊙:

(4) $${\tilde{F}}_{RGB}^{\left(2\right)}=F_{RGB}^{\left(2\right)}⊙s_{RGB},\;{\tilde{F}}_{IR}^{\left(2\right)}=F_{IR}^{\left(2\right)}⊙s_{IR}$$

To reconcile spatial discrepancies, we perform local spatial refinement. Because the dual-branch backbone processes modalities independently, they may retain distinct local spatial distortions. Inspired by standard depthwise separable convolutions [32], the local spatial structures are refined as follows:

(5) $$F_{dw,RGB}=\phi \left({\mathrm{BN}}_{d}\left(K_{d}*{\tilde{F}}_{RGB}^{\left(2\right)}\right)\right),\;F_{dw,IR}=\phi \left({\mathrm{BN}}_{d}\left(K_{d}*{\tilde{F}}_{IR}^{\left(2\right)}\right)\right)$$

(6) $$F_{geo,RGB}^{\left(2\right)}=K_{p}*F_{dw,RGB},\;F_{geo,IR}^{\left(2\right)}=K_{p}*F_{dw,IR}$$

where $K_{d}\in R^{C_{2}\times 1\times 3\times 3}$ is a depthwise convolution kernel, $K_{p}\in R^{C_{2}\times C_{2}\times 1\times 1}$ is a pointwise convolution kernel, ∗ denotes the standard convolution operation, BN(·) denotes BatchNorm, and ϕ(·) is the SiLU activation. The geometrically calibrated features are then concatenated along the channel dimension:

(7) $$Z^{\left(2\right)}=\mathrm{Concat}\left(F_{geo,RGB}^{\left(2\right)},F_{geo,IR}^{\left(2\right)}\right)\in R^{2C_{2}\times H_{2}\times W_{2}}$$

Finally, a convolution layer compresses the dimension back to $C_{2}$:

(8) $$F_{SMC}^{\left(2\right)}=\phi \left({\mathrm{BN}}_{f}\left(K_{f}*Z^{\left(2\right)}\right)\right)$$

where $K_{f}\in R^{C_{2}\times 2C_{2}\times 1\times 1}$. This output dimension matches the input shape $C_{2}\times H_{2}\times W_{2}$, completing the shallow nonlinear fusion process.

#### 3.3.2. Cross-Modal Interaction at Mid-to-High Levels

In multimodal object detection, shallow features (such as P2 and P3) focus primarily on local geometries, edges, and textures, which can be calibrated by convolutions with strong local inductive biases (as performed in the SMC module) without incurring excessive computational overhead. However, high-level semantic and contextual representations are mainly handled by mid-to-high level features such as P4 and P5, as shown in Figure 1. At these semantic levels, relying solely on basic convolution-based fusion (e.g., feature concatenation or element-wise addition) makes it difficult to capture the complementary global context between modalities [33,34]. Furthermore, applying cross-attention at shallow layers is computationally expensive due to the quadratic complexity $O({\left(H\times W\right)}^{2})$ of the attention mechanism.

To address this, we introduce the Cross-Modal Attention (CMA) mechanism specifically at the highly compressed P4 and P5 scales. This design models the long-range dependencies between RGB and IR to achieve semantic complementarity while controlling computational costs, aligning with hierarchical feature interaction strategies proposed for specialized detection tasks [35]. Inspired by cross-modal attention mechanisms in multimodal transformers [36], we construct a bidirectional cross-modal attention module: the RGB branch queries the IR features, and the IR branch queries the RGB features.

In the CMA module, let the input features at layer *l* (l∈{4,5}) be $F_{RGB}^{\left(l\right)},F_{IR}^{\left(l\right)}\in R^{C_{l}\times H_{l}\times W_{l}}$. To perform attention operations, we first flatten the spatial dimensions to obtain sequence representations $X_{RGB},X_{IR}\in R^{N\times C_{l}}$, where $N=H_{l}\times W_{l}$ represents the sequence length.

RGB queries IR: The RGB modality acts as the query, retrieving relevant global context from the infrared modality. The queries ($Q_{RGB}$), keys ($K_{IR}$), and values ($V_{IR}$) are generated through learnable linear projections:

(9) $$Q_{RGB}=X_{RGB}W_{Q}^{RGB},\;K_{IR}=X_{IR}W_{K}^{IR},\;V_{IR}=X_{IR}W_{V}^{IR}$$

where $W_{Q}^{RGB},W_{K}^{IR},W_{V}^{IR}\in R^{C_{l}\times d}$ are the linear projection weight matrices. We set the projection dimension *d* to be equal to the input channel dimension $C_{l}$ ($d=C_{l}$). The cross-modal attention weights are calculated based on dot-product similarity, and the complementary features are obtained through weighted summation:

(10) $$A_{RGB\to IR}^{\left(l\right)}=\mathrm{Softmax}\left(\frac{Q_{RGB}K_{IR}^{⊤}}{\sqrt{d}}\right)V_{IR}$$

where $\sqrt{d}$ is the scaling factor to prevent vanishing gradients. The attention output is passed through a linear projection, added to the original sequence via a residual connection, and then processed by a Multi-Layer Perceptron (MLP) block and Layer Normalization:

(11) $${\tilde{F}}_{RGB}=X_{RGB}+\mathrm{Linear}\left(A_{RGB\to IR}^{\left(l\right)}\right)$$

(12) $$F_{RGB}^{\prime }=\mathrm{LayerNorm}\left({\tilde{F}}_{RGB}+\mathrm{MLP}\left({\tilde{F}}_{RGB}\right)\right)$$

The output $F_{RGB}^{\prime }$ is then reshaped back to the spatial dimensions $R^{C_{l}\times H_{l}\times W_{l}}$.

**Figure 3:**
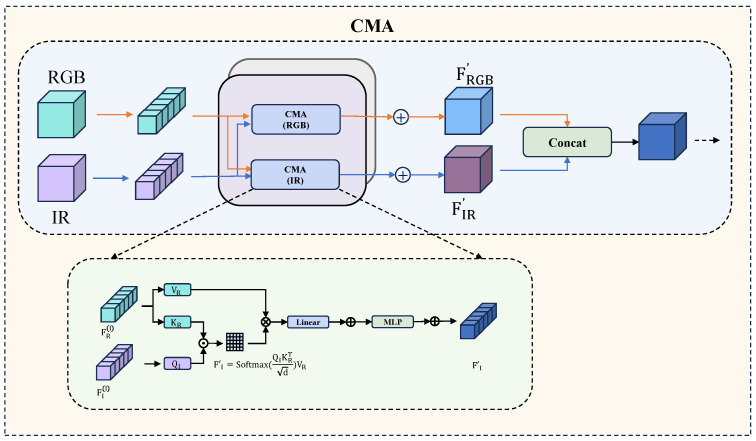
CMA structure diagram. Note that the ⊕ symbol denotes element-wise addition representing the residual connection.

IR queries RGB: Similarly, the infrared modality acts as the query to obtain complementary visual texture information from the RGB modality. As detailed in the zoomed-in block of Figure 3, this dual interaction process is formulated symmetrically:

(13) $$Q_{IR}=X_{IR}W_{Q}^{IR},\;K_{RGB}=X_{RGB}W_{K}^{RGB},\;V_{RGB}=X_{RGB}W_{V}^{RGB}$$

(14) $$A_{IR\to RGB}^{\left(l\right)}=\mathrm{Softmax}\left(\frac{Q_{IR}K_{RGB}^{⊤}}{\sqrt{d}}\right)V_{RGB}$$

(15) $${\tilde{F}}_{IR}=X_{IR}+\mathrm{Linear}\left(A_{IR\to RGB}^{\left(l\right)}\right)$$

(16) $$F_{IR}^{\prime }=\mathrm{LayerNorm}\left({\tilde{F}}_{IR}+\mathrm{MLP}\left({\tilde{F}}_{IR}\right)\right)$$

After the bidirectional interaction, the enhanced representations $F_{RGB}^{\prime }$ and $F_{IR}^{\prime }$ are concatenated along the channel dimension and compressed back to $C_{l}$ channels via a 1×1 convolution, completing the mid-to-high level feature fusion.

#### 3.3.3. Multi-Scale Fusion Feature Enhancement (MFEE)

**Figure 4:**
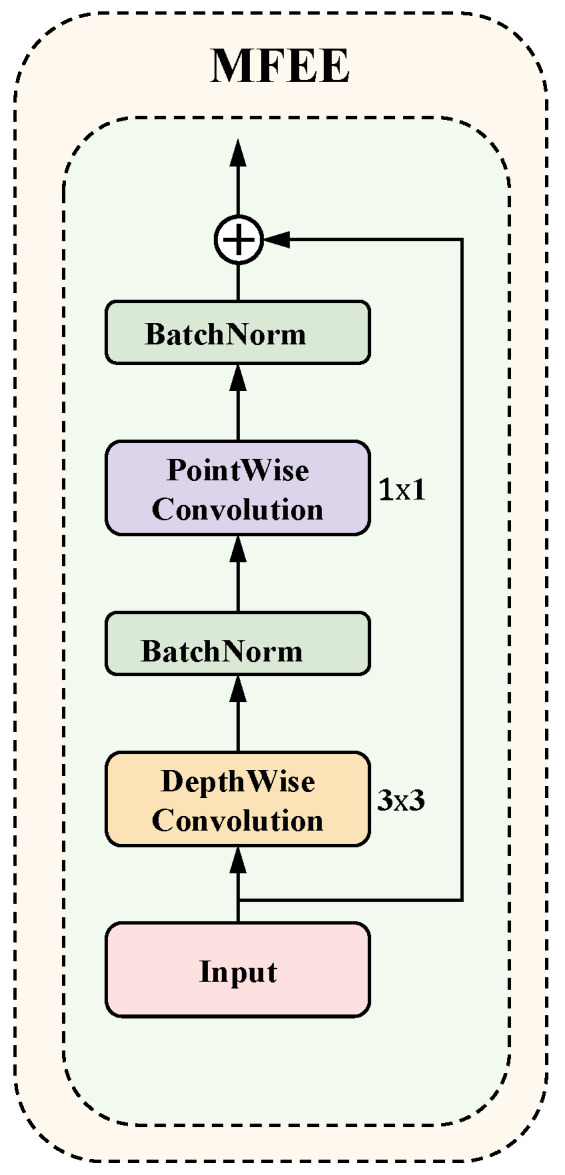
MFEE structure diagram.

In multispectral object detection, improving the discriminative power of fused features is essential for precise localization. Conventional feature concatenation and progressive propagation often introduce redundant background noise and exhibit limited sensitivity to small or distant targets. To address these challenges, we propose the Multi-Scale Fusion Feature Enhancement (MFEE) module, as illustrated in Figure 4. This module refines the fused multispectral representations through a residual enhancement structure, improving feature expressiveness and providing stable inputs for the subsequent Small-Object-Sensitive Detection Head (SOS-Head) and Soft Attention Regularization (SAR-AP) modules.

Feature Refinement Process: Let the fused feature at scale *l* be denoted as $F_{fused}^{\left(l\right)}\in R^{C_{l}\times H_{l}\times W_{l}}$. The enhanced representation $F^{\left(l\right)}$ is formulated as a standard residual addition:

(17) $$F^{\left(l\right)}=H^{\left(l\right)}\left(F_{fused}^{\left(l\right)}\right)+F_{fused}^{\left(l\right)}$$

where $H^{\left(l\right)}\left(\cdot \right)$ represents the refinement sub-network composed of a depthwise spatial convolution and a subsequent 1×1 pointwise convolution. This bottleneck-like design models local spatial correlations and cross-channel interactions independently. The identity shortcut connection preserves the original multimodal fusion context while integrating the refined semantic details. This residual refinement design further enhances the expressiveness of fused multispectral features and provides stable feature inputs for subsequent detection modules.

### 3.4. Soft Attention Regularization Based on Attention Prior (SAR-AP)

**Figure 5:**
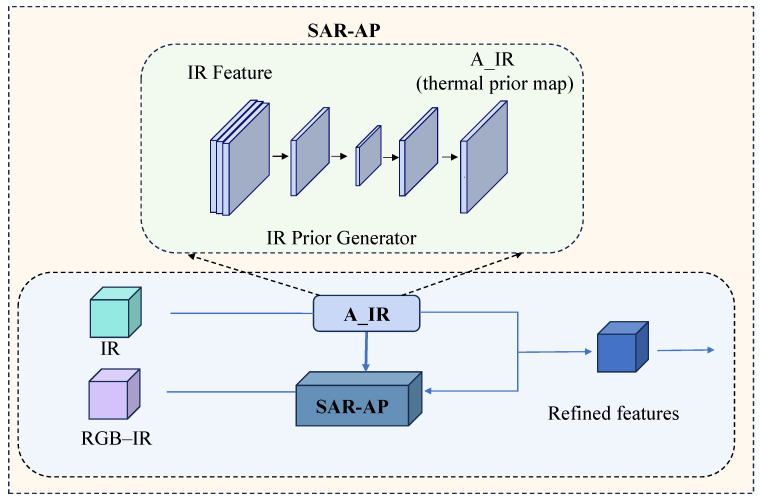
Architecture of the Soft Attention Regularization based on Attention Prior (SAR-AP) module.

To guide the network to focus on critical target regions and suppress attention drift in complex environments, we introduce the SAR-AP module. As illustrated in Figure 5, this module operates in two sequential phases: generating a thermal prior map and applying it as a soft constraint on the fused multimodal features.

It should be noted that the structural consistency considered in SAR-AP is different from conventional modality alignment. Conventional alignment mainly aims to reduce pixel-level, region-level, or feature-level discrepancies between modalities, such as spatial registration or offset-based feature matching. In contrast, SAR-AP does not explicitly estimate geometric offsets, warp feature maps, or force RGB and IR features to share identical representations. Instead, it uses an infrared-derived attention prior to softly regularize the spatial response pattern of fused features. In this work, structural consistency refers to the stability, continuity, and target-region coherence of fused attention responses under dense small-object or background-interference scenarios. In this way, SAR-AP helps reduce attention drift while preserving cross-modal complementarity.

#### 3.4.1. Infrared Prior Generation

The selection of an appropriate prior source is important for regulating cross-modal attention responses. While RGB features provide rich textural details, they remain sensitive to illumination variations. Similarly, generic mathematical priors, such as statistical or saliency-based maps, may not provide sufficient target-specific physical cues. In contrast, infrared thermal radiation offers a stable spatial cue that remains consistent across varying lighting conditions. Consequently, we extract the prior map directly from the infrared modality to serve as a reliable spatial reference.

Let the infrared feature at scale l∈{3,4,5} be $F_{IR}^{\left(l\right)}\in R^{C_{l}\times H_{l}\times W_{l}}$. The infrared prior map $A_{IR}^{\left(l\right)}$ is generated through a prior generator $\Phi_{\mathrm{prior}}^{\left(l\right)}\left(\cdot \right)$, formulated as

(18) $$A_{IR}^{\left(l\right)}=\sigma \left({\mathrm{Conv}}_{1\times 1}\left(\phi \left(\mathrm{BN}\left({\mathrm{Conv}}_{3\times 3}\left(F_{IR}^{\left(l\right)}\right)\right)\right)\right)\right)$$

where $A_{IR}^{\left(l\right)}\in {\left[0,1\right]}^{1\times H_{l}\times W_{l}}$ represents the spatial saliency of thermal targets, BN(·) denotes Batch Normalization, ϕ(·) is the SiLU (Sigmoid Linear Unit) activation function, and σ(·) represents the Sigmoid function used to constrain the spatial weights to the [0,1] range.

#### 3.4.2. Soft Regularization and Loss Formulation

The fused feature $F^{\left(l\right)}$ generates its own attention map $A^{\left(l\right)}\in {\left[0,1\right]}^{1\times H_{l}\times W_{l}}$. To regularize the spatial response pattern of $A^{\left(l\right)}$ using the infrared prior $A_{IR}^{\left(l\right)}$ without over-constraining the network’s representational capacity, we introduce a soft regularization term. The $L_{2}$ norm is selected as the constraint metric based on the following mathematical considerations:

- Comparison with KL-Divergence: KL-divergence requires the inputs to be spatial probability distributions (summing to 1). However, our attention maps are generated via Sigmoid activations, representing independent pixel-wise saliency rather than a joint distribution.
- Comparison with $L_{1}$ Norm and Cosine Similarity: The $L_{1}$ norm tends to yield sparse and rigid gradients, which can destabilize the training of multi-branch networks. Meanwhile, cosine similarity only measures directional similarity, ignoring the absolute activation magnitude (energy) of the attention features.

Since the infrared prior $A_{IR}^{\left(l\right)}$ often contains some thermal background noise, the $L_{2}$ norm acts as a smooth, soft penalty. It enables the network to learn a general target-related spatial response pattern while preserving the flexibility of multimodal feature learning [37]. The multi-scale attention regularization loss is defined as

(19) $$L_{SAR}=\sum_{l\in \{3,4,5\}}\alpha_{l}{∥A^{\left(l\right)}-A_{IR}^{\left(l\right)}∥}_{2}^{2}$$

where $\alpha_{l}$ are scale-specific weights. To prioritize the protection of small objects, which are highly susceptible to attention drift, these coefficients are empirically set to assign a higher penalty to the relatively high-resolution P3 branch among the SAR-AP layers, thereby focusing the regularization on fine-grained target-related response regions.

### 3.5. Small-Object-Sensitive Detection Head (SOS-Head)

In multispectral object detection, small objects—such as distant pedestrians and vehicles—occupy limited pixel areas and are susceptible to information loss during successive down-sampling stages. Standard YOLO11 detection heads primarily perform predictions at three scales: P3 for medium-sized objects and P4/P5 for larger targets, which often lack explicit modeling for high-resolution small-object features. To address this limitation, we introduce a specialized branch at the shallow P2 layer to preserve fine-grained spatial information.

After being processed by the Dual-Branch Backbone and the CMHF module, the P2 layer yields the highest spatial resolution RGB–IR fused features (160×160 for a 640×640 input). The proposed SOS-Head takes these features as input and establishes a high-resolution detection path. To capture small-object attributes against complex backgrounds while controlling architectural complexity, SOS-Head utilizes Dynamic Convolution coupled with multi-scale feature enhancement [38,39].

Dynamic Kernel Generation: Let the input feature to the dynamic convolution be $x\in R^{C\times H\times W}$. Unlike conventional convolutions with static weights, dynamic convolution adaptively aggregates *K* parallel expert kernels $\left\{W_{1},W_{2},\ldots ,W_{K}\right\}$ based on input-dependent attention weights. These weights are generated by extracting global spatial information via Global Average Pooling (GAP), followed by a compact multi-layer perceptron (MLP) and a Sigmoid activation σ(·):

(20) $$\pi \left(x\right)=\sigma \left(W_{\mathrm{attn}}^{\left(2\right)}\phi \left(W_{\mathrm{attn}}^{\left(1\right)}\mathrm{GAP}\left(x\right)\right)\right)$$

where $\pi \left(x\right)=[\pi_{1}\left(x\right),\pi_{2}\left(x\right),\ldots ,\pi_{K}\left(x\right)]$ represents the dynamically generated routing weights for the *K* experts, ϕ(·) is the ReLU activation, and $W_{\mathrm{attn}}^{\left(1\right)},W_{\mathrm{attn}}^{\left(2\right)}$ denote the learnable weights of the MLP. The aggregated dynamic kernel $W_{\mathrm{dyn}}$ is then formulated as a linear combination of the expert kernels:

(21) $$W_{\mathrm{dyn}}\left(x\right)=\sum_{i=1}^{K}\pi_{i}\left(x\right)W_{i}$$

The output of the dynamic convolution is computed as $y=\psi (\mathrm{BN}\left(W_{\mathrm{dyn}}\left(x\right)*x\right))$, where ∗ denotes the convolution operation, BN(·) is Batch Normalization, and ψ(·) is the activation function. This mechanism allows the network to adaptively adjust its receptive field and feature extraction focus based on the specific distribution of small objects in the current scene.

**Figure 6:**
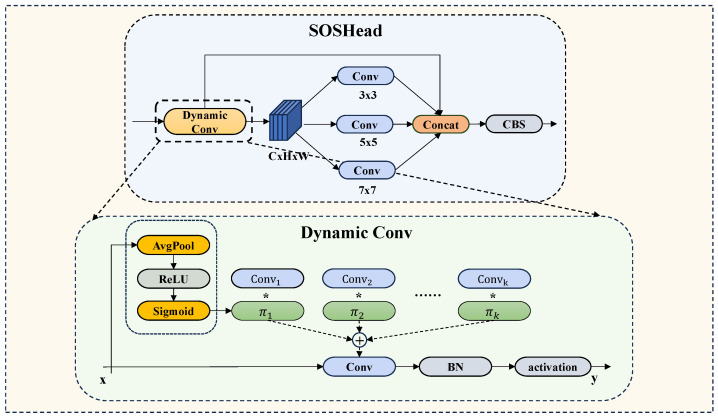
SOS -Head structure diagram.

Following the dynamic convolution, the features are processed through a multi-scale structure. As illustrated in Figure 6, three parallel branches with kernel sizes of 3×3, 5×5, and 7×7 are utilized to extract features across different receptive fields. These multi-scale representations are concatenated and fused through a final Conv-BN-SiLU (CBS) block. By combining high-resolution P2 features with adaptive multi-scale convolution, SOS-Head strengthens the representation and localization of small targets without relying solely on deeper low-resolution feature maps.

## 4. Experiments

### 4.1. Dataset

#### 4.1.1. Dataset Details

The experiments in this study are conducted on three publicly available multispectral object detection benchmarks: M3FD [40], VEDAI [41], and LLVIP [42].

M3FD Dataset: The detection subset of the M3FD benchmark comprises 4200 registered RGB and infrared (IR) image pairs. It contains 33,603 annotated object instances across six categories: People, Car, Bus, Motorcycle, Truck, and Lamp. This dataset primarily features stationary monitoring and in-vehicle perception scenarios. We follow the official benchmark protocols for data partitioning and evaluation. Both RGB and IR streams are utilized as dual-modal inputs to evaluate the proposed MCF-YOLO framework.

VEDAI Dataset: The VEDAI (Vehicle Detection in Aerial Imagery) dataset focuses on small-object detection in remote sensing imagery. It consists of 1210 registered RGB and IR image pairs, where we perform recognition on over 3700 annotated object instances across seven vehicle categories. Captured from a top-down satellite perspective, the targets in this dataset exhibit very small spatial scales. We follow the official benchmark protocols for data partitioning and evaluation. In this study, we train the MCF-YOLO framework from scratch on this dataset to evaluate its effectiveness under aerial viewpoints.

LLVIP Dataset: The LLVIP benchmark focuses on low-light pedestrian detection, containing 15,488 aligned RGB and IR image pairs captured across 24 nighttime and 2 daytime scenes. We follow the official benchmark protocols for data partitioning and evaluation. We utilize this dataset to evaluate the zero-shot cross-dataset generalization capability of the model on the pedestrian category. Specifically, the MCF-YOLO framework, trained on the M3FD dataset, is directly evaluated on the official LLVIP validation set without any fine-tuning, thereby assessing its cross-dataset generalization under domain shifts.

#### 4.1.2. Data Preprocessing and Modality Alignment

All RGB–IR image pairs used in this study are directly obtained from the official datasets, and the original pairing and registration relationships provided by each benchmark are preserved. No additional manual alignment, image registration, or extra cropping operation is applied before training or testing. For each paired sample, the RGB image and the corresponding infrared image are loaded according to the official pairing relationship and shared annotations, ensuring one-to-one correspondence between the two modalities.

During preprocessing, both RGB and infrared images are resized to the same network input resolution. The same resizing scale and padding strategy are applied to the two modalities to maintain their spatial correspondence after preprocessing. In this study, no additional offline data augmentation or synthetic sample generation is introduced beyond the standard training pipeline. For online data augmentation used during training, such as Mosaic augmentation and other geometric transformations, the same random transformation parameters are applied synchronously to paired RGB and IR images. This strategy avoids spatial misalignment caused by independent modality transformations and preserves the paired structure of multispectral inputs. Following the training setting, Mosaic augmentation is disabled during the last 10 epochs to allow the model to further optimize bounding box localization on images closer to the original data distribution.

#### 4.1.3. Dataset Analysis and Motivation

**Figure 7:**
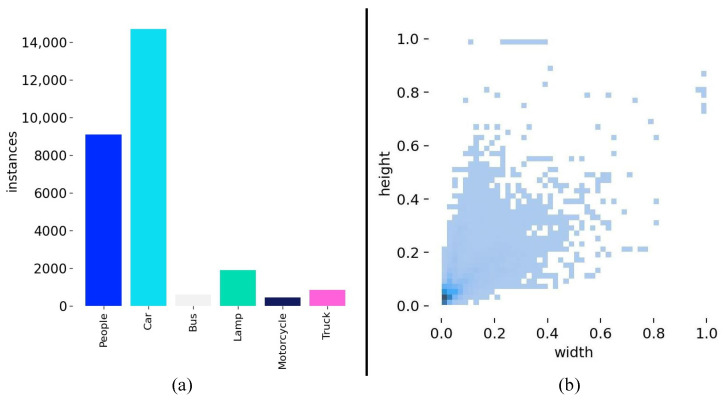
Statistical analysis of the M3FD dataset annotations. (**a**) Category distribution exhibiting a long-tail characteristic with class imbalance. (**b**) Normalized object size distribution (width vs. height) highlighting the high concentration of small objects.

To analyze the characteristics of the M3FD dataset and motivate the design of the Small-Object-Sensitive Detection Head (SOS-Head), we evaluate the dataset annotations, as shown in Figure 7.

As illustrated in Figure 7a, the dataset exhibits a long-tail distribution. The Car and People categories constitute the majority of instances, presenting a class imbalance that challenges detection precision for minority classes. The normalized object size distribution in Figure 7b shows that a large proportion of targets are concentrated in the small-width and small-height regions. This indicates that many targets, such as distant pedestrians and minute vehicles, occupy limited pixel areas. In standard YOLO11 pipelines, the spatial information of such targets is often degraded during repeated down-sampling operations. Consequently, the SOS-Head is designed to leverage high-resolution P2 features to preserve spatial details for these small-scale objects.

To evaluate the generalization capability of the proposed architecture beyond the M3FD distribution, we incorporate the VEDAI and LLVIP datasets for specific evaluation scenarios.

The VEDAI dataset provides an aerial-view detection scenario, where targets are generally small due to the top-down imaging perspective. This dataset is used to evaluate the small-object representation capability of the proposed architecture. While the M3FD analysis justifies the need for small-object sensitivity in ground-level scenarios, VEDAI provides a stricter scale challenge. We utilize this dataset for ablation studies and comparative evaluations to test the capability of the SOS-Head in preserving spatial features under different viewing angles.

The LLVIP dataset is used to validate the cross-dataset generalization of the proposed method. We perform zero-shot evaluation on LLVIP using the model trained on M3FD. Featuring low-light conditions with degraded visible features, this evaluation assesses whether the learned multispectral representations remain effective when visible textures are degraded.

### 4.2. Implementation Details

#### 4.2.1. Experimental Setup and Hyperparameters

The experiments were conducted under the Ubuntu 22.04 environment. The detailed hardware configuration and software specifications are summarized in Table 1. All model training and inference procedures were executed on the same workstation to ensure consistent evaluation conditions.

During the training phase, the proposed MCF-YOLO network was optimized using Stochastic Gradient Descent (SGD) for 200 epochs. The input image resolution was standardized to 640×640 pixels. The initial learning rate was set to 0.01, with a momentum factor of 0.937 and a weight decay of 0.0005. The batch size was set to 16, and 4 data-loading workers were used. The hyperparameter settings are detailed in Table 2.

To refine bounding-box localization during the final convergence stage, Mosaic augmentation was disabled during the last 10 epochs (close_mosaic = 10). This strategy allows the model to fine-tune its spatial prediction capability on images closer to the original data distribution after the initial heavy augmentation phase.

All baseline, ablation, and comparison experiments with executable implementations were conducted under identical hardware and hyperparameter settings to ensure reproducibility and fairness of the comparative evaluation.

For inference efficiency evaluation, all FPS values reported in the comparison tables were measured under the same RTX 4090-based PyTorch inference environment for models with executable implementations. To reduce the influence of CUDA initialization and runtime fluctuation, each model was first warmed up for 100 iterations. The subsequent speed evaluation was conducted on 1000 images with a batch size of 1. CUDA synchronization was performed before and after timing, and the average FPS was calculated according to the total inference time. Therefore, the reported FPS values reflect the relative inference efficiency of different models under the same controlled software and hardware settings.

#### 4.2.2. Evaluation Metrics

To quantitatively evaluate the detection performance and computational complexity of the proposed model, we adopt the following standard metrics: Precision, Recall, Mean Average Precision (mAP), Giga Floating-point Operations (GFLOPs), and the number of parameters. The mAP is reported both at a single Intersection over Union (IoU) threshold of 0.5 (mAP@0.5) and averaged across IoU thresholds from 0.5 to 0.95 (mAP@0.5:0.95).

(1) Precision and Recall

Precision measures the proportion of correctly detected targets in the prediction results, while Recall measures the proportion of correctly detected targets out of all true targets. Their definitions are as follows:

(22) $$\mathrm{Precision}=\frac{TP}{TP+FP}\;\mathrm{Recall}=\frac{TP}{TP+FN}$$

where True Positive (TP) represents the number of correctly detected targets, False Positive (FP) represents the number of false detections, and False Negative (FN) represents the number of missed detections.

(2) Mean Average Precision (mAP)

mAP is a standard performance metric in object detection tasks, used to evaluate the model’s detection accuracy across different classes.

The average precision (AP) is calculated for each class, and then the mean of all the classes is taken:

(23) $$\mathrm{mAP}=\frac{1}{N}\sum_{i=1}^{N}AP_{i}$$

where *N* is the total number of classes and $AP_{i}$ is the average precision for the *i*-th class.

This paper uses two common metrics: mAP@0.5, calculated at an IoU threshold of 0.5, and mAP@0.5:0.95, averaged over IoU values from 0.5 to 0.95 (with a step size of 0.05), which better reflects the overall stability and reliability of the model.

(3) Giga Floating-point Operations (GFLOPs)

GFLOPs is used to measure the computational complexity of the model during inference, representing the total number of floating-point operations (in billions) required to process a single input.

(4) Number of Parameters

The number of parameters is used to measure the model’s size, representing the total number of trainable parameters in the model.

(5) Frames Per Second (FPS)

FPS is used to measure the inference efficiency of the model, indicating the number of images processed per second during inference. It is defined as follows:

(24) $$\mathrm{FPS}=\frac{N_{\mathrm{img}}}{T_{\mathrm{infer}}}$$

where $N_{\mathrm{img}}$ denotes the number of input images and $T_{\mathrm{infer}}$ denotes the total inference time. A higher FPS indicates higher inference efficiency.

(6) Size-Weighted Precision and Recall

To evaluate the practical application value of the model more fairly for extreme small-object detection and other challenging scenarios, we introduce a size-weighted, double-summation evaluation system. Traditional metrics often aggregate predictions across entire datasets, obscuring performance on individual challenging images containing tiny or blurred objects. To directly address the limitations of the conventional metrics and provide a fairer comparison, we improve the expressions into an image-level double-summation format.

Let *M* denote the total number of test images. For the *i*-th image, let $N_{i}$ be the number of ground-truth objects. We explicitly define the size-weighted True Positives (${\mathrm{TP}}_{i}^{w}$) and False Negatives (${\mathrm{FN}}_{i}^{w}$) for each image using an indicator function I(·):

(25) $${\mathrm{TP}}_{i}^{w}=\sum_{j=1}^{N_{i}}w_{i,j}I\left(g_{i,j}\in \mathrm{TP}\right)\;{\mathrm{FN}}_{i}^{w}=\sum_{j=1}^{N_{i}}w_{i,j}I\left(g_{i,j}\in \mathrm{FN}\right)$$

where $g_{i,j}$ represents the *j*-th ground-truth object in the *i*-th image. To emphasize the detection difficulty of smaller targets while avoiding weight explosion, the object-size weight $w_{i,j}$ is mathematically defined with image-level normalization:

(26) $$w_{i,j}=\frac{{\left(a_{i,j}+\epsilon \right)}^{-\gamma }}{\frac{1}{N_{i}}\sum_{t=1}^{N_{i}}{\left(a_{i,t}+\epsilon \right)}^{-\gamma }}$$

where $a_{i,j}$ is the normalized bounding box area, γ controls the intensity of the weight applied to small objects, and ϵ is a small constant for numerical stability.

Crucially, since false positives (FP) represent incorrect predictions without corresponding ground-truth objects, they lack natural size weights. Therefore, we apply a unit penalty for each false alarm, defining ${\mathrm{FP}}_{i}^{w}={\mathrm{FP}}_{i}$. The final size-weighted Precision and Recall across the dataset are computed via the outer summation over *M* images:

(27) $${\mathrm{Precision}}_{w}=\frac{1}{M}\sum_{i=1}^{M}\frac{{\mathrm{TP}}_{i}^{w}}{{\mathrm{TP}}_{i}^{w}+{\mathrm{FP}}_{i}+\epsilon }\;{\mathrm{Recall}}_{w}=\frac{1}{M}\sum_{i=1}^{M}\frac{{\mathrm{TP}}_{i}^{w}}{{\mathrm{TP}}_{i}^{w}+{\mathrm{FN}}_{i}^{w}+\epsilon }$$

(7) Size-Weighted Mean Average Precision (${\mathrm{mAP}}_{w}$)

Based on the image-level double-summation Precision and Recall defined above, we further reformulate the conventional mAP into a size-weighted form. This modification directly extends the object-size weighting mechanism from fixed-threshold evaluation to threshold-varying AP calculation.

While size-weighted Precision and Recall evaluate the model at a fixed confidence threshold, comprehensively assessing a detector requires calculating the Average Precision (AP) across all possible thresholds.

For a specific object category *c* and a given Intersection over Union (IoU) threshold τ, varying the confidence score threshold *s* yields a continuous set of size-weighted precision and recall values, thereby forming a size-weighted Precision–Recall (PR) curve. The size-weighted Average Precision ($AP_{w}^{c,\tau }$) is defined as the area under this curve:

(28) $$AP_{w}^{c,\tau }=\int_{0}^{1}{\mathrm{Precision}}_{w}^{c,\tau }\left(R\right)\;dR$$

where ${\mathrm{Precision}}_{w}^{c,\tau }\left(R\right)$ represents the size-weighted precision at a specific size-weighted Recall level *R*.

To evaluate the model’s overall detection capability across the dataset, the size-weighted mean Average Precision (${\mathrm{mAP}}_{w}^{\tau }$) at a specific IoU threshold τ is computed by taking the mean of the AP across all *C* categories:

(29) $${\mathrm{mAP}}_{w}^{\tau }=\frac{1}{C}\sum_{c=1}^{C}AP_{w}^{c,\tau }$$

To provide a holistic evaluation that reflects both classification accuracy and the strictness of bounding-box spatial localization, particularly for small objects, we adopt two primary variations of this comprehensive metric:

${\mathrm{mAP}}_{w}@0.5$: The size-weighted mAP calculated at a single, standard IoU threshold of τ=0.5.

${\mathrm{mAP}}_{w}@0.5\text{:}0.95$: The size-weighted mAP averaged over 10 distinct IoU thresholds ranging from 0.5 to 0.95 with a step size of 0.05. This formulation follows the standard mAP@0.5:0.95 evaluation protocol while incorporating the proposed size-aware weighting mechanism:

(30) $${\mathrm{mAP}}_{w}@0.5\text{:}0.95=\frac{1}{10}\sum_{\tau \in \{0.5,0.55,\ldots ,0.95\}}{\mathrm{mAP}}_{w}^{\tau }$$

### 4.3. Comparison Experiments

To evaluate the overall performance and cross-scenario generalization of the proposed MCF-YOLO, comparative experiments are conducted on both the M3FD and VEDAI datasets. M3FD is used to evaluate general multispectral detection performance in ground-level traffic and surveillance scenarios, while VEDAI is used to further examine small-object detection capability under aerial viewpoints. As detailed in Table 3 and Table 4, the evaluated baselines include standard single-modality networks and representative multispectral fusion architectures.

To ensure experimental consistency for reproducible methods, open-source models with available implementations were reproduced and trained from scratch under the same data splits, hardware environment, and optimization strategy. For methods without available executable implementations or complete evaluation results in our environment, the results from their original publications are cited and unavailable metrics are denoted as “–”.

Among the single-modality networks, YOLOv8n (RGB) achieves the highest mAP@0.5 on M3FD, reaching 76.9%. However, constructing a dual-stream architecture based on this backbone would increase the computational burden, as YOLOv8n requires 8.09 GFLOPs per stream. In contrast, YOLO11n requires 6.32 GFLOPs and 2.6M parameters. Given the feature extraction requirements of multimodal architectures, YOLO11n is selected as a suitable foundational backbone. Overall, the results in Table 3 and Table 4 show that well-designed multimodal detectors can achieve stronger detection performance than single-modality detectors, indicating that RGB–IR feature fusion is beneficial under both ground-level and aerial-view evaluation settings.

On the M3FD dataset, the proposed MCF-YOLO achieves an mAP@0.5 of 82.6%, outperforming the Mid-fusion baseline by 2.7 percentage points. More importantly, under the stricter mAP@0.5:0.95 metric, MCF-YOLO reaches 54.9%, surpassing representative multispectral detectors such as ICAFusion (50.8%) and MOD-YOLO-Tiny (50.9%). This indicates that the proposed cross-modal fusion strategy and SOS-Head contribute to improved bounding-box localization. Regarding model complexity, MCF-YOLO requires 4.8M parameters, maintaining a smaller model size than high-performing models such as ICAFusion (5.9M) and MOD-YOLO-Tiny (16.0M). Although MCF-YOLO introduces higher GFLOPs than some basic fusion baselines, it achieves higher detection accuracy and stricter localization performance with a moderate parameter scale.

On the VEDAI dataset, MCF-YOLO also achieves the highest mAP@0.5 and mAP@0.5:0.95, reaching 72.9% and 45.0%, respectively. Compared with representative fusion architectures such as DEYOLO (71.6% mAP@0.5) and SuperYOLO (71.1% mAP@0.5), the proposed method demonstrates improved detection performance under aerial viewpoints. In addition, MCF-YOLO achieves the highest Precision of 77.2%, indicating that the proposed fusion strategy helps reduce false detections in complex aerial backgrounds. These results are consistent with the design motivation of SAR-AP and SOS-Head, which aim to suppress irrelevant responses and enhance small-object localization.

**Figure 8:**
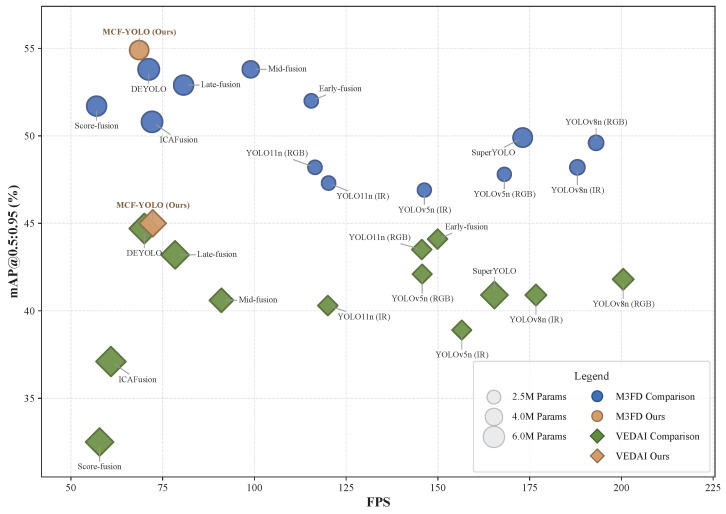
Accuracy–efficiency trade-off comparison on the M3FD and VEDAI datasets. The x-axis denotes inference speed (FPS), the y-axis denotes mAP@0.5:0.95, and the bubble size represents the number of parameters. Different markers distinguish results on M3FD and VEDAI, and the proposed MCF-YOLO is highlighted separately. Only models with available FPS and mAP@0.5:0.95 results are included.

From the perspective of inference efficiency, single-modality detectors generally achieve higher FPS because they process only a single input stream. However, their detection accuracy is generally lower than that of the best-performing multimodal methods, especially under the stricter mAP@0.5:0.95 metric. As shown in Figure 8, MCF-YOLO achieves the highest mAP@0.5:0.95 on both M3FD and VEDAI among the models with available FPS measurements. Although its FPS is lower than that of some single-modality detectors and SuperYOLO, it maintains acceptable inference efficiency while achieving stronger localization performance. Since TarDAL, MOD-YOLO-Tiny, FID-YOLO, and YOLOrs lack either FPS or mAP@0.5:0.95 results, they are not included in the scatter plot.

### 4.4. Ablation Experiments

This section systematically evaluates the contributions of the proposed MCF-YOLO framework. Component ablation experiments are first conducted on the M3FD and VEDAI datasets to examine the individual and combined effects of CMHF, SAR-AP, and SOS-Head. Then, module-level comparisons are performed to distinguish the proposed components from functionally related designs in existing multispectral detection methods. Qualitative visualizations are further provided to analyze the effect of SOS-Head on small-object detection and to clarify the response-level structural consistency introduced by SAR-AP. Finally, micro-ablation studies and category-wise AP analysis are conducted to explain the internal design choices and the performance variations across different object categories and scale distributions.

#### 4.4.1. Component Ablation on M3FD and VEDAI

To evaluate the effectiveness and individual contributions of the proposed modules—CMHF, SAR-AP, and SOS-Head—we conduct ablation experiments on the M3FD and VEDAI datasets. M3FD represents ground-level multispectral detection scenarios with mixed object scales, while VEDAI provides an aerial-view scenario dominated by small objects. The quantitative results are summarized in Table 5 and Table 6, respectively.

As shown in Table 5 and Table 6, the independent introduction of a single module does not always lead to stable improvements. When CMHF is used alone, the mAP@0.5 on M3FD slightly decreases from 79.9% to 79.7%, while on VEDAI the mAP@0.5 improves from 67.5% to 69.1% but Recall drops from 68.7% to 60.6%. This indicates that hierarchical cross-modal interaction alone may introduce unstable responses without sufficient feature regularization. SAR-AP brings moderate improvements on M3FD, increasing mAP@0.5 from 79.9% to 80.5%, but its independent effect on VEDAI remains limited. This suggests that the infrared-guided prior can help suppress background interference, but spatial prior constraints alone are insufficient for aerial scenes with small targets and complex textures.

The results of SOS-Head further show that high-resolution detection depends on the quality of the preceding fused features. On M3FD, using SOS-Head alone increases Precision from 84.1% to 89.1%, but Recall and mAP@0.5:0.95 decrease, indicating that high-resolution spatial details alone do not guarantee better overall detection. This limitation is more evident on VEDAI, where the independent use of SOS-Head reduces mAP@0.5 from 67.5% to 54.7%. In contrast, when SOS-Head is combined with SAR-AP, the VEDAI mAP@0.5 increases to 71.3%, suggesting that small-object-sensitive detection becomes more effective when high-resolution features are regularized by attention-prior guidance.

The complete MCF-YOLO model achieves the best overall performance on both datasets. Compared with the baseline, it improves mAP@0.5 from 79.9% to 82.6% and mAP@0.5:0.95 from 53.8% to 54.9% on M3FD. On VEDAI, it increases mAP@0.5 from 67.5% to 72.9% and mAP@0.5:0.95 from 40.6% to 45.0%. These results demonstrate that CMHF, SAR-AP, and SOS-Head are complementary rather than independently additive. CMHF provides multi-scale cross-modal interaction, SAR-AP regularizes fused responses, and SOS-Head enhances small-object localization based on more stable high-resolution features. Although the complete model introduces additional computation, it achieves a reasonable accuracy–complexity trade-off in challenging multispectral detection scenarios.

#### 4.4.2. Comparison with Related Functional Modules

To further distinguish the proposed components from closely related designs, we conduct module- and design-level replacement experiments under the same baseline framework. Specifically, each related module or functional design is integrated into the corresponding position of the baseline, while the remaining network components, training settings, input resolution, and dataset split are kept unchanged within each comparison group. For methods without a clearly separated standalone module, we implement or adapt their core functional design according to the original description. This comparison is intended to evaluate the functional differences between related module designs rather than to compare complete detection frameworks.

Table 7 compares CMHF and SAR-AP with functionally related fusion and attention/alignment designs on the M3FD dataset using standard detection metrics. In the fusion-design comparison, CMHF achieves the best Recall, mAP@0.5, and mAP@0.5:0.95, while obtaining the same Precision as DACFusion. This suggests that hierarchical cross-modal feature interaction across multiple semantic levels can preserve complementary RGB–IR information more effectively than single-stage or locally designed fusion operations.

In the attention/alignment comparison, SAR-AP achieves the best results across all metrics. Among the compared methods, RFA and DeformCAT are included as representative alignment-related designs. Unlike these alignment-oriented designs or purely feature-driven attention mechanisms, SAR-AP introduces an infrared-guided soft attention prior to regularize the fused spatial response pattern. Therefore, its main role is to improve response-level structural consistency rather than to perform direct pixel-level or feature-level alignment between RGB and IR modalities.

To further evaluate the effectiveness of SOS-Head for small-object enhancement, we compare it with several representative small-object enhancement designs using the proposed size-weighted metrics. As shown in Table 8, SOS-Head achieves the best results across all four metrics, including ${\mathrm{Precision}}_{w}$, ${\mathrm{Recall}}_{w}$, ${\mathrm{mAP}}_{w}@0.5$, and ${\mathrm{mAP}}_{w}@0.5\text{:}0.95$. This indicates that SOS-Head provides better small-object-sensitive detection performance under an evaluation protocol that assigns larger weights to smaller ground-truth objects.

Compared with the AGLC-YOLO design, SOS-Head improves ${\mathrm{Precision}}_{w}$, ${\mathrm{Recall}}_{w}$, ${\mathrm{mAP}}_{w}@0.5$, and ${\mathrm{mAP}}_{w}@0.5\text{:}0.95$ by 6.83, 6.71, 8.49, and 6.33 percentage points, respectively. Compared with the PCPE-YOLO design, the corresponding improvements are 1.42, 0.34, 1.31, and 1.82 percentage points. SOS-Head also outperforms the YOLO-SSP and ES-YOLOv8 designs on all four metrics. In particular, although YOLO-SSP and ES-YOLOv8 obtain competitive ${\mathrm{mAP}}_{w}@0.5$, SOS-Head further improves ${\mathrm{mAP}}_{w}@0.5\text{:}0.95$ to 36.18%, suggesting better localization stability under stricter IoU thresholds.

These results show that the dedicated high-resolution branch and multi-scale convolutional design of SOS-Head are more effective in preserving fine-grained spatial cues for small targets. Unlike generic small-object enhancement designs that mainly improve feature representation in a general manner, SOS-Head directly strengthens the high-resolution detection pathway. Therefore, it provides more balanced improvements in weighted recall and weighted mAP, demonstrating its suitability for small-object-sensitive RGB–IR detection.

#### 4.4.3. Qualitative Ablation and Structural Interpretation

**Figure 9:**
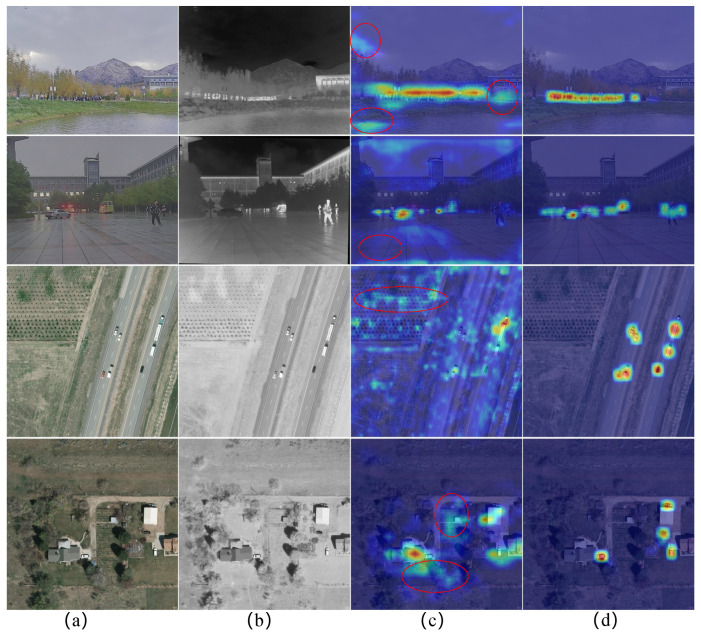
Visualization comparison of activation responses with and without SAR-AP. Columns (**a**–**d**) denote the RGB image, IR image, heatmap without SAR-AP, and heatmap with SAR-AP, respectively. The first two rows are from M3FD, and the last two rows are from VEDAI. Red dashed ellipses highlight scattered background activations produced without SAR-AP.

To clarify the role of SAR-AP, we visualize the activation responses of the models with and without SAR-AP on representative samples from M3FD and VEDAI. As shown in Figure 9, the heatmaps without SAR-AP in column (c) show scattered or over-expanded activations in non-target background regions, which are highlighted by the red dashed ellipses. These responses appear around roadsides, vegetation, road structures, buildings, and other cluttered background areas, indicating that the fused features are easily affected by background interference when no attention-prior regularization is applied. In contrast, after introducing SAR-AP, the heatmaps in column (d) become more compact and target-oriented, with stronger responses concentrated around pedestrians, vehicles, or thermally salient target regions.

These observations indicate that SAR-AP does not explicitly perform geometric alignment between RGB and IR features. Instead, it regularizes the fused response distribution to reduce background-induced activation drift. In this work, structural consistency refers to the spatial coherence and target-oriented compactness of fused responses, rather than pixel-level or feature-level alignment between modalities.

**Figure 10:**
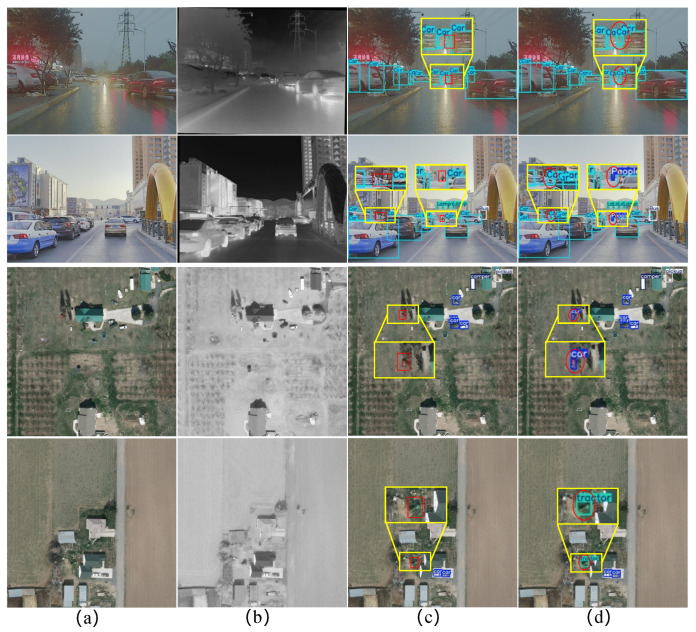
Qualitative comparison of detection results with and without SOS-Head. Columns (**a**–**d**) denote the RGB image, IR image, detection results without SOS-Head, and detection results of the complete MCF-YOLO, respectively. The first two rows are from M3FD, and the last two rows are from VEDAI. Yellow enlarged regions highlight representative small or distant targets. Red dashed rectangles indicate missed or weakly detected targets without SOS-Head, while ellipses indicate the corresponding targets detected by MCF-YOLO.

To qualitatively evaluate SOS-Head, we compare the detection results of the model without SOS-Head and the complete MCF-YOLO. As shown in Figure 10, removing SOS-Head leads to missed or weak detections for small and distant targets, especially in crowded traffic scenes and aerial-view images where objects occupy only a limited number of pixels. After introducing SOS-Head, the corresponding targets are more reliably detected in the highlighted regions.

This improvement is visible in both M3FD and VEDAI scenarios. In M3FD, SOS-Head helps recover distant vehicles and pedestrians under complex traffic backgrounds. In VEDAI, it improves the detection of tiny aerial targets against cluttered ground textures. These results suggest that the high-resolution P2 branch helps preserve fine-grained spatial details, thereby improving small-object sensitivity in multispectral detection.

#### 4.4.4. Micro-Ablation of Internal Mechanisms

To validate the structural rationale of the internal designs, we conduct micro-ablation studies on the SAR-AP and SOS-Head modules. The experiments are evaluated on the M3FD dataset.

Table 9 details the performance of different constraint functions and prior sources. Among the constraint types, while the $L_{1}$ norm achieves the highest precision (88.0%), its overall mAP@0.5 is lower. Probabilistic constraints (KL Divergence) and geometric constraints (Cosine Similarity) also yield lower accuracy. The $L_{2}$ norm performs best overall, as it provides an appropriate soft penalty that aligns spatial semantics without excessively suppressing overlapping cross-modal features.

Regarding the prior sources, replacing the infrared modality prior with mathematical representations (Fixed, Saliency, or Prediction Priors) degrades performance, indicating a lack of specific target semantics required for complex scenarios. Notably, while the RGB prior achieves a comparable mAP@0.5 to the IR prior, its performance under the strict mAP@0.5:0.95 metric drops to 53.4%. This reflects the physical instability of visible light features under poor illumination or occlusion. The infrared prior provides a consistent spatial reference, making the combination of the IR prior and $L_{2}$ loss an effective configuration for feature modulation.

To evaluate the effectiveness of SOS-Head under the proposed size-weighted metrics, we replace it with several representative feature aggregation necks and detection heads while keeping the same CMHF+SAR-AP framework. As shown in Table 10, DyHead achieves the highest ${\mathrm{Precision}}_{w}$ of 73.05% and a slightly higher ${\mathrm{mAP}}_{w}@0.5\text{:}0.95$ of 36.21%, which are higher than those of SOS-Head by 3.45 and 0.03 percentage points, respectively. This may be attributed to its attention-based head design, which tends to improve prediction selectivity and localization quality under stricter IoU thresholds. However, its ${\mathrm{Recall}}_{w}$ is lower than that of SOS-Head, indicating that some small or difficult targets may still be missed.

In contrast, SOS-Head achieves the highest ${\mathrm{Recall}}_{w}$ of 71.66% and the best ${\mathrm{mAP}}_{w}@0.5$ of 56.70%. Compared with LSKNet, Gold-YOLO, DyHead, and BiFPN, SOS-Head improves ${\mathrm{Recall}}_{w}$ by 4.44, 3.25, 1.93, and 3.62 percentage points, respectively. It also improves ${\mathrm{mAP}}_{w}@0.5$ by 3.12, 2.81, 0.45, and 2.43 percentage points, respectively. These results indicate that SOS-Head is more effective in reducing missed detections of small objects under the size-weighted evaluation. Although its ${\mathrm{Precision}}_{w}$ and ${\mathrm{mAP}}_{w}@0.5\text{:}0.95$ are slightly lower than those of DyHead, the dedicated high-resolution branch and multi-scale convolutional design provide stronger small-object recall and more balanced weighted mAP performance, demonstrating its suitability for small-object-sensitive RGB–IR detection.

#### 4.4.5. Category-Wise Analysis and Scale-Related Trade-Offs

To investigate the category-specific effects of the proposed architecture, particularly the SOS-Head, we report the per-class size-weighted Average Precision ${\mathrm{AP}}_{w}@0.5$ on both the M3FD and VEDAI datasets in Table 11 and Table 12, respectively. This metric is used as a supplementary indicator to further examine the performance differences across categories with different object scales and imaging characteristics.

The single-modality baselines show clear modality-dependent characteristics under the size-weighted evaluation. On M3FD, the IR model performs better than the RGB model on the Peoplecategory, while the RGB model achieves much higher ${\mathrm{AP}}_{w}@0.5$ on Lamp. This indicates that infrared images are more effective for thermally salient targets under weak illumination, whereas visible images provide more discriminative texture cues for visually distinctive but thermally weak objects. Multispectral fusion alleviates these modality-specific limitations and improves the overall ${\mathrm{mAP}}_{w}@0.5$.

After introducing SOS-Head, MCF-YOLO achieves the highest overall ${\mathrm{mAP}}_{w}@0.5$ on both datasets. On M3FD, compared with the model without SOS-Head, the overall ${\mathrm{mAP}}_{w}@0.5$ improves from 56.43% to 56.70%. Specifically, People, Car, Lamp, and Truck improve by 2.15, 1.00, 3.89, and 0.53 percentage points, respectively. These gains indicate that the high-resolution P2 branch in SOS-Head helps preserve fine-grained spatial cues for small or weakly textured targets. However, Bus and Motorcycle decrease by 2.26 and 3.66 percentage points, respectively, suggesting that the benefit of high-resolution enhancement varies across categories and may be affected by object scale, appearance ambiguity, and background complexity.

On VEDAI, the effect of SOS-Head becomes more evident under the size-weighted evaluation due to the aerial viewpoint and the dominance of small objects. Compared with the model without SOS-Head, MCF-YOLO improves the overall ${\mathrm{mAP}}_{w}@0.5$ from 39.92% to 45.00%. In particular, Car, Pickup, Camper, and Plane improve by 14.72, 17.59, 5.31, and 14.41 percentage points, respectively. These improvements show that SOS-Head is beneficial for small aerial targets with limited spatial details. In contrast, Truck, Tractor, and Boat decrease by 0.85, 6.07, and 4.45 percentage points, respectively, which may be related to category-specific appearance ambiguity and scale distribution in aerial imagery. Overall, the category-wise size-weighted results show that SOS-Head improves small-object-sensitive detection performance, while its benefits vary across categories depending on object scale, background complexity, and modality complementarity.

### 4.5. Zero-Shot Cross-Dataset Generalization Evaluation

To evaluate the transferability of the proposed architecture in low-light environments, we conduct a zero-shot cross-dataset evaluation on the LLVIP dataset. Specifically, all models are trained only on the M3FD dataset and then directly evaluated on the LLVIP validation set without any fine-tuning or domain adaptation. Since LLVIP contains pedestrian annotations, the evaluation is conducted on the pedestrian category. The quantitative results are reported in Table 13.

As shown by the single-modality baselines, YOLO11n achieves an mAP@0.5 of 27.2% using RGB images and 32.0% using IR images. The low zero-shot performance indicates a clear domain gap between M3FD and LLVIP, especially because LLVIP mainly contains low-light and nighttime pedestrian scenes. Compared with single-modality models, multimodal fusion methods generally improve zero-shot performance. For example, the Mid-fusion baseline reaches an mAP@0.5 of 38.7%, showing that RGB–IR fusion provides more transferable representations under unseen low-light conditions.

**Figure 11:**
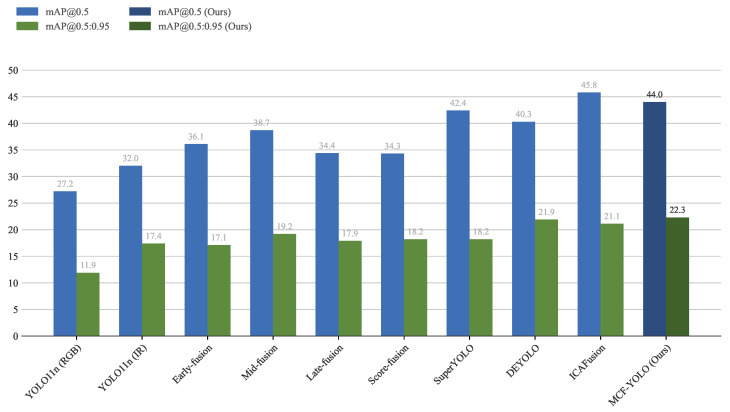
Zero-shot cross-dataset generalization comparison on the LLVIP validation set. All models are trained solely on M3FD and tested directly on LLVIP without fine-tuning or domain adaptation. The grouped bars show mAP@0.5 and mAP@0.5:0.95, with MCF-YOLO highlighted for visual comparison.

Among the evaluated multimodal models, ICAFusion achieves the highest Recall of 52.2% and the highest mAP@0.5 of 45.8%, as reported in Table 13. Figure 11 further visualizes the comparison of mAP@0.5 and mAP@0.5:0.95. Compared with ICAFusion, MCF-YOLO obtains higher Precision, increasing from 56.7% to 60.9%, and achieves the best mAP@0.5:0.95 of 22.3%. Although MCF-YOLO does not obtain the highest Recall or mAP@0.5, its stronger performance under the stricter IoU metric suggests better localization robustness in the zero-shot low-light setting. These results indicate that the proposed architecture maintains competitive cross-dataset generalization while providing improved localization quality under domain shift.

### 4.6. Visualization Analysis

**Figure 12:**
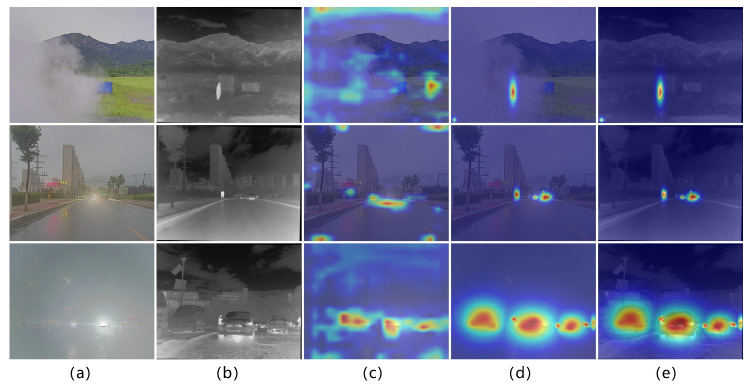
Grad-CAM visualization in complex RGB– IR scenarios. Columns (**a**–**e**) denote the RGB image, IR image, heatmap of YOLO11-RGB, heatmap of the MCF-YOLO infrared branch, and heatmap of the final fused feature response of MCF-YOLO, respectively. The selected scenes include dense smoke, heavy fog, and nighttime glare.

To further analyze the qualitative behavior of MCF-YOLO in challenging RGB–IR scenarios, we employ Grad-CAM to visualize feature activation heatmaps. For fair comparison, all heatmaps are generated using the same Grad-CAM settings and normalization strategy. As shown in Figure 12, three representative scenes with dense smoke, heavy fog, and nighttime glare are selected. These scenes contain distant or small targets and therefore provide challenging cases for evaluating feature response stability.

The RGB-only baseline in Figure 12c produces dispersed and weak activations under degraded visible conditions, with responses drifting toward background regions such as smoke, fog, and road reflections. In contrast, the infrared branch of MCF-YOLO in Figure 12d provides more stable responses around thermally salient targets. After cross-modal fusion, the final fused feature response of MCF-YOLO in Figure 12e shows more compact and target-oriented activation regions. These results indicate that MCF-YOLO can exploit infrared cues to reduce background-induced response drift and enhance target-related feature responses under complex imaging conditions.

**Figure 13:**
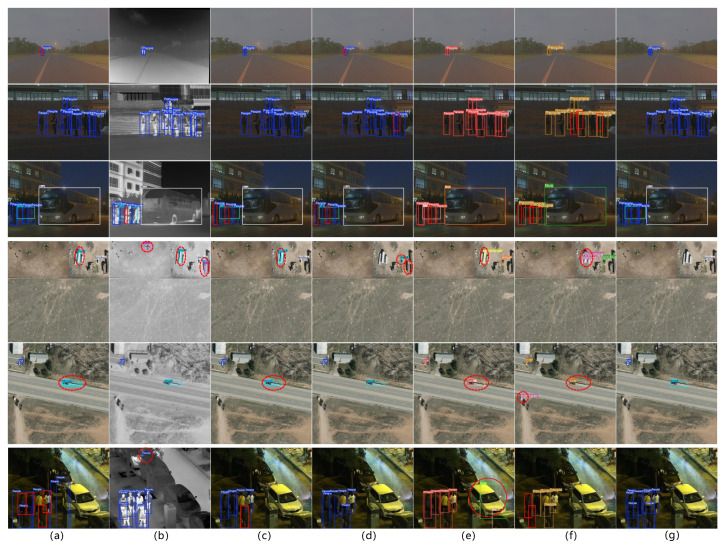
Qualitative detection comparison across multiple datasets. Columns (**a**–**g**) denote YOLO11-RGB, YOLO11-IR, Mid-fusion, ICAFusion, DEYOLO, SuperYOLO, and MCF-YOLO, respectively. Rows 1–3 are from M3FD, rows 4–5 are from VEDAI, and row 6 is from LLVIP. Red dashed boxes or ellipses highlight representative missed detections, false detections, misclassifications, or inaccurate bounding boxes in comparison methods.

To further evaluate the qualitative detection performance, Figure 13 compares detection results across M3FD, VEDAI, and LLVIP. The comparison includes single-modality baselines, basic fusion baselines, and representative multispectral detection methods. Rows 1–3 are from M3FD, rows 4–5 are from VEDAI, and row 6 is from LLVIP.

As shown in Figure 13, comparison methods may suffer from missed detections, false detections, or inaccurate bounding boxes under modality degradation, aerial viewpoints, and dense low-light pedestrian scenes. In M3FD scenes, visible degradation and background interference cause some methods to miss thermally salient targets or generate unstable predictions. In VEDAI scenes, small aerial targets and cluttered backgrounds lead to misclassifications or incomplete bounding boxes. In the LLVIP zero-shot scene, dense pedestrians and overlapping thermal responses make it difficult to separate adjacent targets. In these representative examples, MCF-YOLO produces more stable detection results, with fewer missed targets and more compact bounding boxes. These qualitative results are consistent with the quantitative improvements in mAP@0.5:0.95 and support the effectiveness of the proposed fusion and small-object-sensitive detection design.

### 4.7. Failure Case Analysis

**Figure 14:**
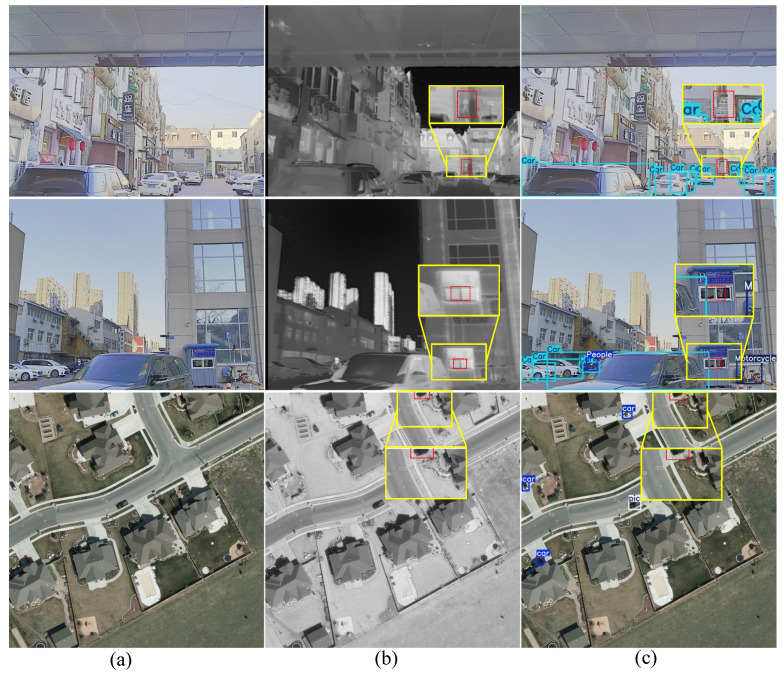
Representative failure cases of MCF-YOLO. Columns (**a**–**c**) denote the RGB image, IR image, and detection result, respectively. Yellow enlarged regions highlight challenging areas, and red dashed boxes indicate failure-prone regions. Rows 1–2 are selected from M3FD, showing distant or occluded targets and reflection-induced ambiguity in complex urban scenes. Row 3 is selected from VEDAI, showing extremely small aerial targets near image boundaries or cluttered backgrounds.

Although MCF-YOLO improves multispectral feature fusion and small-object detection, several failure cases still remain, as shown in Figure 14. In the first row, the targets are distant and partially occluded by surrounding vehicles, buildings, and street-side structures. Since these targets occupy only a few pixels and are mixed with dense urban background patterns, the model may produce unstable localization or confuse them with nearby object-like structures. This indicates that distant small targets under partial occlusion remain challenging even with multispectral fusion.

The second row shows a reflection-related failure case. In the RGB image, the glass facade and reflective surfaces introduce strong visual interference. In the IR image, the corresponding region presents weak or ambiguous thermal responses rather than a clear target-like thermal structure. As a result, the model may incorrectly respond to reflective or thermally ambiguous regions, leading to false detections. This suggests that strong reflections and abnormal thermal responses can still disturb semantic discrimination.

The third row illustrates a failure case from the VEDAI dataset. The vehicle is extremely small and located close to the image boundary, where only limited contextual information is available. In this situation, road boundaries, rooftops, shadows, and surrounding ground textures may interfere with localization, causing missed detections, false positives, or unstable bounding boxes. These observations help explain the category-wise performance fluctuations reported in Table 12, where some aerial categories show decreased AP under scale variation and background ambiguity. Therefore, better handling of occluded targets, reflection suppression, thermal ambiguity modeling, and boundary-aware small-object localization remain important directions for future work.

## 5. Conclusions

Built upon the YOLO11 architecture, this paper proposes MCF-YOLO, a multi-level cross-modality fusion network for RGB–IR object detection. The proposed Cross-Modal Hierarchical Fusion (CMHF) framework progressively enhances multispectral feature interaction across different network stages. Specifically, shallow cross-modal calibration is used to reduce low-level modality inconsistency, mid-level interaction strengthens semantic complementarity, and deep fused feature enhancement improves multi-scale representation. In addition, SAR-AP introduces an infrared-derived attention prior to regularize fused feature responses in a soft manner, thereby reducing background-induced response drift and improving response-level structural consistency. Furthermore, SOS-Head is introduced on the high-resolution P2 branch to enhance small-object sensitivity and improve the localization of small or distant targets.

Extensive experiments on the M3FD and VEDAI datasets demonstrate the effectiveness of the proposed method. MCF-YOLO achieves the best mAP@0.5 and mAP@0.5:0.95 on both datasets among the compared models, with 4.8M parameters and 17.88 GFLOPs. In terms of inference efficiency, MCF-YOLO obtains 68.6 FPS on M3FD and 72.3 FPS on VEDAI under the same RTX 4090-based PyTorch testing environment, showing a reasonable balance between detection accuracy and inference efficiency. In addition, the zero-shot evaluation on LLVIP further indicates that MCF-YOLO maintains competitive cross-dataset generalization and achieves the best mAP@0.5:0.95 under low-light domain shift.

Despite these improvements, several limitations remain. The model may still produce missed detections or unstable localization under severe occlusion, strong specular reflections, ambiguous thermal responses, cross-modal spatial inconsistency, and extremely small or boundary-truncated targets. In addition, the current efficiency evaluation is conducted on a high-performance GPU, and empirical deployment on physical edge devices has not yet been performed. Future work will focus on better handling of occluded targets, reflection suppression, thermal ambiguity modeling, and boundary-aware small-object localization. We will also investigate hardware-oriented optimization and deployment strategies, such as pruning, knowledge distillation, INT8 quantization, and TensorRT acceleration, to further evaluate the practical applicability of MCF-YOLO on edge computing platforms.

## Figures and Tables

**Table 1:** System environment and hardware configuration.

Configuration Item	Specification
CPU	Intel(R) Xeon(R) Gold 5418Y
GPU	NVIDIA GeForce RTX 4090 (24 GB)
Memory	120 GB RAM
Operating System	Ubuntu 22.04
Language	Python 3.10
Framework	PyTorch 2.3.1
Computing Platform	CUDA 12.1

**Table 2:** Hyperparameter settings for the training phase.

Hyperparameter	Value
Input Resolution	640×640
Batch Size	16
Data Workers	4
Total Training Epochs	200
Optimizer	SGD
Initial Learning Rate	0.01
Momentum	0.937
Weight Decay	0.0005
Mosaic Augmentation	Closed last 10 epochs

**Table 3:** Experimental comparison of different models on the M3FD dataset. The best values for each metric are highlighted in bold.

Index	Model	Data	Precision	Recall	mAP@0.5	mAP@0.5:0.95	Params	GFLOPs	FPS
Single-modality networks									
1	YOLOv5n	RGB	81.9%	65.3%	74.1%	47.8%	**2.5M**	7.07	168.1
2	YOLOv5n	IR	85.7%	61.7%	71.8%	46.9%	**2.5M**	7.07	146.3
3	YOLOv8n	RGB	84.8%	69.0%	76.9%	49.6%	3.0M	8.09	**193.1**
4	YOLOv8n	IR	81.5%	65.5%	73.2%	48.2%	3.0M	8.09	188.0
5	YOLO11n	RGB	81.6%	65.9%	73.5%	48.2%	2.6M	**6.32**	116.5
6	YOLO11n	IR	84.8%	61.3%	71.5%	47.3%	2.6M	**6.32**	120.2
Multi-modality networks									
7	Early-fusion	RGB + IR	83.8%	69.7%	78.5%	52.0%	2.6M	6.35	115.5
8	Mid-fusion	RGB + IR	84.1%	73.4%	79.9%	53.8%	3.8M	9.31	99.0
9	Late-fusion	RGB + IR	82.9%	73.1%	80.0%	52.9%	5.2M	12.31	80.7
10	Score-fusion	RGB + IR	84.3%	69.6%	78.6%	51.7%	5.2M	12.68	57.0
11	TarDAL [40]	RGB + IR	–	–	80.7%	–	7.1M	14.88	–
12	SuperYOLO [43]	RGB + IR	80.4%	70.1%	78.0%	49.9%	4.9M	56.60	173.1
13	DEYOLO [44]	RGB + IR	86.8%	72.9%	81.2%	53.8%	6.0M	8.41	71.2
14	ICAFusion [45]	RGB + IR	85.5%	72.5%	82.1%	50.8%	5.9M	13.91	72.1
15	MOD-YOLO-Tiny [46]	RGB + IR	**87.1%**	72.7%	81.8%	50.9%	16.0M	–	–
16	FID-YOLO [47]	RGB + IR	–	–	80.3%	53.5%	8.1M	22.40	–
–	MCF-YOLO (Ours)	RGB + IR	**87.1%**	**73.7%**	**82.6%**	**54.9%**	4.8M	17.88	68.6

**Table 4:** Experimental comparison of different models on the VEDAI dataset. The best values for each metric are highlighted in bold.

Index	Model	Data	Precision	Recall	mAP@0.5	mAP@0.5:0.95	Params	GFLOPs	FPS
Single-modality networks									
1	YOLOv5n	RGB	49.8%	**81.9%**	69.7%	42.1%	**2.5M**	7.07	145.7
2	YOLOv5n	IR	55.6%	65.4%	64.6%	38.9%	**2.5M**	7.07	156.5
3	YOLOv8n	RGB	60.3%	68.0%	66.2%	41.8%	3.0M	8.09	**200.5**
4	YOLOv8n	IR	50.8%	67.9%	66.6%	40.9%	3.0M	8.09	176.7
5	YOLO11n	RGB	71.3%	60.7%	67.1%	43.5%	2.6M	**6.32**	145.6
6	YOLO11n	IR	69.1%	51.9%	63.7%	40.3%	2.6M	**6.32**	120.0
Multi-modality networks									
7	Early-fusion	RGB + IR	58.7%	66.6%	66.5%	44.1%	2.6M	6.35	149.9
8	Mid-fusion	RGB + IR	55.3%	68.7%	67.5%	40.6%	3.8M	9.31	91.0
9	Late-fusion	RGB + IR	67.4%	69.6%	71.4%	43.2%	5.2M	12.31	78.4
10	Score-fusion	RGB + IR	49.6%	67.3%	55.6%	32.5%	5.2M	12.68	57.8
11	YOLOrs [48]	RGB + IR	41.4%	76.6%	59.7%	–	–	–	–
12	SuperYOLO	RGB + IR	63.8%	67.1%	71.1%	40.9%	4.9M	56.60	165.4
13	DEYOLO	RGB + IR	68.7%	53.1%	71.6%	44.7%	6.0M	8.41	70.0
14	ICAFusion	RGB + IR	71.3%	65.9%	69.0%	37.1%	5.9M	13.91	60.9
15	MOD-YOLO-Tiny	RGB + IR	–	–	58.4%	34.6%	16.0M	–	–
–	MCF-YOLO (Ours)	RGB + IR	**77.2%**	65.1%	**72.9%**	**45.0%**	4.8M	17.88	72.3

**Table 5:** Quantitative ablation results of the proposed framework components on the M3FD dataset. The baseline (Exp. 1) is a dual-branch mid-fusion network. The checkmark (✓) indicates the inclusion of the module. Best results are in bold.

Exp.	CMHF	SAR-AP	SOS-Head	Precision	Recall	mAP@0.5	mAP@0.5:0.95	GFLOPs	Params
1				84.1%	73.4%	79.9%	53.8%	**9.31**	**3.8M**
2	✓			86.9%	72.1%	79.7%	53.5%	17.14	4.5M
3		✓		85.4%	73.4%	80.5%	53.9%	17.44	4.7M
4			✓	**89.1%**	70.8%	80.2%	53.3%	18.33	4.7M
5	✓	✓		84.9%	73.1%	80.5%	54.0%	17.65	4.6M
6	✓		✓	86.8%	71.0%	79.5%	53.3%	16.89	4.1M
7		✓	✓	84.1%	73.6%	80.5%	54.2%	17.02	4.6M
8	✓	✓	✓	87.1%	**73.7%**	**82.6%**	**54.9%**	17.88	4.8M

**Table 6:** Quantitative ablation results on the VEDAI dataset. The baseline (Exp. 1) is a dual-branch mid-fusion network. Best results are in bold.

Exp.	CMHF	SAR-AP	SOS-Head	Precision	Recall	mAP@0.5	mAP@0.5:0.95	GFLOPs	Params
1				55.3%	68.7%	67.5%	40.6%	**9.31**	**3.8M**
2	✓			63.4%	60.6%	69.1%	39.4%	17.14	4.5M
3		✓		64.9%	64.7%	65.0%	39.1%	17.44	4.7M
4			✓	69.6%	54.6%	54.7%	34.8%	18.33	4.7M
5	✓	✓		68.0%	65.0%	70.0%	41.1%	17.65	4.6M
6	✓		✓	64.5%	52.7%	58.2%	37.3%	16.89	4.1M
7		✓	✓	69.4%	**68.8%**	71.3%	42.3%	17.02	4.6M
8	✓	✓	✓	**77.2%**	65.1%	**72.9%**	**45.0%**	17.88	4.8M

**Table 7:** Module-level comparison of the proposed CMHF and SAR-AP with related fusion and attention/alignment designs on the M3FD dataset. All compared designs are integrated into the same baseline framework and trained under identical settings. The best value within each comparison group is highlighted in bold.

Component/Design	Precision	Recall	mAP@0.5	mAP@0.5:0.95
Fusion design comparison for CMHF				
DACFusion [17]	**86.9%**	70.3%	79.4%	52.3%
EI2Det [18]	84.2%	71.0%	78.0%	52.0%
CLFM [19]	81.6%	69.3%	75.3%	50.1%
**CMHF (Ours)**	**86.9%**	**72.1%**	**79.7%**	**53.5%**
Attention/alignment design comparison for SAR-AP				
RFA [20]	83.7%	69.6%	76.7%	51.2%
DeformCAT [21]	84.4%	69.1%	77.1%	50.6%
EAEF [22]	83.8%	71.3%	78.9%	53.0%
T-aware [23]	84.3%	68.7%	77.3%	51.5%
Target-aware [24]	80.1%	70.3%	77.6%	51.8%
**SAR-AP (Ours)**	**85.4%**	**73.4%**	**80.5%**	**53.9%**

**Table 8:** Comparison of small-object enhancement designs on the M3FD dataset using the proposed size-weighted metrics. All compared designs are integrated into the same baseline framework and trained under identical settings. The best value for each metric is highlighted in bold.

Component/Design	Precision_*w*_	Recall_*w*_	mAP_*w*_@0.5	mAP_*w*_@0.5:0.95
AGLC-YOLO design [25]	62.77%	64.95%	48.21%	29.85%
PCPE-YOLO design [26]	68.18%	71.32%	55.39%	34.36%
YOLO-SSP design [27]	68.24%	70.87%	56.52%	34.60%
ES-YOLOv8 design [28]	68.38%	70.60%	56.02%	34.65%
**SOS-Head (Ours)**	**69.60%**	**71.66%**	**56.70%**	**36.18%**

**Table 9:** Ablation study on the internal design of the feature modulation module (SAR-AP). All variants are built upon the same CMHF+SAR-AP framework without the SOS-Head, and only the constraint function or prior source in SAR-AP is changed. Best results are highlighted in bold.

Model Variations	Precision	Recall	mAP@0.5	mAP@0.5:0.95
Constraint Type (with IR Prior)				
w/KL Divergence	85.4%	70.8%	80.1%	53.5%
w/$L_{1}$ Loss	**88.0%**	71.7%	80.2%	53.5%
w/Cosine Similarity	86.7%	71.2%	79.4%	53.8%
Prior Source (with $L_{2}$ Loss)				
w/RGB Prior	86.8%	71.7%	**80.5%**	53.4%
w/Fixed Prior	85.4%	71.2%	78.6%	52.3%
w/Saliency Prior	86.3%	72.4%	79.3%	53.0%
w/Statistical Prior	85.1%	70.6%	79.3%	**54.0%**
w/Prediction Prior	85.2%	72.1%	79.2%	52.1%
**Ours ($L_{2}$ Loss + IR Prior)**	84.9%	**73.1%**	**80.5%**	**54.0%**

**Table 10:** Comparison of different feature aggregation necks and detection heads under the proposed size-weighted metrics. All variants are evaluated within the same CMHF+SAR-AP framework on the M3FD dataset. The best value for each metric is highlighted in bold.

Module	${\mathrm{Precision}}_{w}$	${\mathrm{Recall}}_{w}$	${\mathrm{mAP}}_{w}@0.5$	${\mathrm{mAP}}_{w}@0.5\text{:}0.95$
w/LSKNet	68.26%	67.22%	53.58%	35.14%
w/Gold-YOLO	70.63%	68.41%	53.89%	34.72%
w/DyHead	**73.05%**	69.73%	56.25%	**36.21%**
w/BiFPN	71.15%	68.04%	54.27%	34.44%
**Ours (SOS-Head)**	69.60%	**71.66%**	**56.70%**	36.18%

**Table 11:** Comparison of per-class ${\mathrm{AP}}_{w}@0.5$ on the M3FD dataset. Best results among the multispectral fusion variants are highlighted in bold.

Model	${\mathrm{mAP}}_{w}@0.5$	People	Car	Bus	Lamp	Motorcycle	Truck
Single Modality Baselines							
YOLO11-RGB	48.49%	42.96%	80.38%	63.74%	38.79%	23.87%	41.21%
YOLO11-IR	48.03%	57.56%	76.90%	66.87%	21.47%	21.35%	44.05%
Multispectral Fusion Variants							
Baseline (Naive Mid-fusion)	55.21%	56.83%	81.88%	**71.31%**	40.32%	28.15%	**52.74%**
Ours w/o SOS-Head	56.43%	60.95%	82.28%	68.57%	46.77%	**32.49%**	47.52%
**MCF-YOLO**	**56.70%**	**63.10%**	**83.28%**	66.31%	**50.66%**	28.83%	48.05%

**Table 12:** Comparison of per-class ${\mathrm{AP}}_{w}@0.5$ on the VEDAI dataset. Best results among the multispectral fusion variants are highlighted in bold.

Model	${\mathrm{mAP}}_{w}@0.5$	Car	Truck	Pickup	Tractor	Camper	Boat	Plane
Single Modality Baselines								
YOLO11-RGB	37.12%	52.72%	21.63%	33.76%	15.82%	47.34%	8.46%	17.21%
YOLO11-IR	25.95%	49.09%	15.00%	28.87%	13.24%	27.60%	11.84%	36.00%
Multispectral Fusion Variants								
Baseline (Naive Mid-fusion)	38.44%	53.51%	**26.62%**	36.44%	27.25%	34.83%	8.18%	20.66%
Ours w/o SOS-Head	39.92%	54.55%	24.45%	35.12%	**35.36%**	31.95%	**19.24%**	18.66%
**MCF-YOLO**	**45.00%**	**69.27%**	23.60%	**52.71%**	29.29%	**37.26%**	14.79%	**33.07%**

**Table 13:** Zero-shot cross-dataset evaluation on the LLVIP validation set. Models are trained solely on M3FD and tested directly on LLVIP without fine-tuning or domain adaptation. The best value for each metric is highlighted in bold.

Index	Model	Data	Precision	Recall	mAP@0.5	mAP@0.5:0.95
Single-modality networks						
1	YOLO11n	RGB	47.9%	25.1%	27.2%	11.9%
2	YOLO11n	IR	**64.0%**	27.4%	32.0%	17.4%
Multi-modality networks						
3	Early-fusion	RGB + IR	57.8%	29.7%	36.1%	17.1%
4	Mid-fusion	RGB + IR	51.3%	36.5%	38.7%	19.2%
5	Late-fusion	RGB + IR	55.3%	35.8%	34.4%	17.9%
6	Score-fusion	RGB + IR	62.7%	29.8%	34.3%	18.2%
7	SuperYOLO	RGB + IR	51.4%	39.4%	42.4%	18.2%
8	DEYOLO	RGB + IR	52.9%	37.5%	40.3%	21.9%
9	ICAFusion	RGB + IR	56.7%	**52.2%**	**45.8%**	21.1%
–	MCF-YOLO (Ours)	RGB + IR	60.9%	39.1%	44.0%	**22.3%**

## Data Availability

Data will be made available on request.

## References

[1] Gallagher J.E., Oughton E.J. (2025). Surveying You Only Look Once (YOLO) Multispectral Object Detection Advancements, Applications, and Challenges. IEEE Access.

[2] Wei J., As’arry A., Rezali K.A.M., Yusoff M.Z.M., Ma H., Zhang K. (2025). A Review of YOLO Algorithm and Its Applications in Autonomous Driving Object Detection. IEEE Access.

[3] Zhang X., Cao S.-Y., Wang F., Zhang R., Wu Z., Zhang X., Bai X., Shen H.-L. (2024). Rethinking Early-Fusion Strategies for Improved Multispectral Object Detection. arXiv.

[4] Sun X., Zhu Y., Huang H. (2025). Specificity-Guided Cross-Modal Feature Reconstruction for RGB-Infrared Object Detection. IEEE Trans. Intell. Transp. Syst..

[5] Shen J., Zhan H., Dong S., Zuo X., Yang W., Ling H. (2025). Multispectral State-Space Feature Fusion: Bridging Shared and Cross-Parametric Interactions for Object Detection. arXiv.

[6] Zhao Y., Gao Y., Yang X., Yang L. (2025). Multispectral Target Detection Based on Deep Feature Fusion of Visible and Infrared Modalities. Appl. Sci..

[7] Tian B., Lu Z., Zhang C., Li H., Yu P. (2025). MSMD-YOLO: Multi-Scale and Multi-Directional Mamba Scanning Infrared Image Object Detection Based on YOLO. Infrared Phys. Technol..

[8] Qiang H., Hao W., Xie M., Tang Q., Shi H., Zhao Y., Han X. (2025). SCM-YOLO for Lightweight Small Object Detection in Remote Sensing Images. Remote Sens..

[9] Wan D., Lu R., Fang Y., Lang X., Shu S., Chen J., Shen S., Xu T., Ye Z. (2025). YOLOv11-RGBT: Towards a Comprehensive Single-Stage Multispectral Object Detection Framework. arXiv.

[10] Redmon J., Divvala S., Girshick R., Farhadi A. You Only Look Once: Unified, Real-Time Object Detection. Proceedings of the IEEE Conference on Computer Vision and Pattern Recognition (CVPR).

[11] Khanam R., Hussain M. (2024). YOLOv11: An Overview of the Key Architectural Enhancements. arXiv.

[12] Zhao T., Yuan M., Jiang F., Wang N., Wei X. (2026). Removal Then Selection: A Coarse-to-Fine Fusion Perspective for RGB-Infrared Object Detection. IEEE Trans. Intell. Transp. Syst..

[13] Zhang H., Fromont É., Lefèvre S., Avignon B. Guided attentive feature fusion for multispectral pedestrian detection. Proceedings of the IEEE Winter Conference on Applications of Computer Vision (WACV).

[14] Liu J., Zhang S., Wang S., Metaxas D.N. Multispectral Deep Neural Networks for Pedestrian Detection. Proceedings of the British Machine Vision Conference (BMVC).

[15] Chen Z., Ji H., Zhang Y. (2026). Global–local feature optimization based RGB–IR fusion object detection on drone view. Chin. J. Aeronaut..

[16] Guo J., Gao C., Liu F., Meng D., Gao X. (2024). DAMSDet: Dynamic Adaptive Multispectral Detection Transformer with Competitive Query Selection and Adaptive Feature Fusion. arXiv.

[17] Qian J., Qiao B., Zhang Y., Liu T., Wang S., Wu G., Han D. (2025). DACFusion: Dual Asymmetric Cross-Attention Guided Feature Fusion for Multispectral Object Detection. Neurocomputing.

[18] Hu K., He Y., Li Y., Zhao J., Chen S., Kang Y. (2025). EI2Det: Edge-Guided Illumination-Aware Interactive Learning for Visible-Infrared Object Detection. IEEE Trans. Circuits Syst. Video Technol..

[19] Peng P., Xu T., Song L., Zhu M., Fang Y., Li J. (2026). COXNet: Cross-Layer Fusion With Adaptive Alignment and Scale Integration for RGBT Tiny Object Detection. IEEE Trans. Circuits Syst. Video Technol..

[20] Zhang L., Zhu X., Chen X., Yang X., Lei Z., Liu Z. Weakly Aligned Cross-Modal Learning for Multispectral Pedestrian Detection. Proceedings of the IEEE/CVF International Conference on Computer Vision (ICCV).

[21] Hu Y., Chen X., Wang S., Liu L., Shi H., Fan L., Tian J., Liang J. (2025). Deformable Cross-Attention Transformer for Weakly Aligned RGB–T Pedestrian Detection. IEEE Trans. Multimed..

[22] Liang M., Hu J., Bao C., Feng H., Deng F., Lam T.L. (2023). Explicit Attention-Enhanced Fusion for RGB-Thermal Perception Tasks. IEEE Robot. Autom. Lett..

[23] Wang H., Song K., Huang L., Wen H., Yan Y. (2023). Thermal Images-Aware Guided Early Fusion Network for Cross-Illumination RGB-T Salient Object Detection. Eng. Appl. Artif. Intell..

[24] Zhang X., Zhang X., Wang J., Ying J., Sheng Z., Yu H., Li C., Shen H.-L. (2025). TFDet: Target-Aware Fusion for RGB-T Pedestrian Detection. IEEE Trans. Neural Netw. Learn. Syst..

[25] Wei L., Han K., Li X., Che H., Hu Z. (2025). Global-Local Context Enhanced YOLO for Small Object Detection in UAV Images. Res. Sq..

[26] Chen W., Liu J., Liu T., Zhuang Y. (2025). PCPE-YOLO with a Lightweight Dynamically Reconfigurable Backbone for Small Object Detection. Sci. Rep..

[27] Liu Y., Yang D., Song T., Ye Y., Zhang X. (2025). YOLO-SSP: An Object Detection Model Based on Pyramid Spatial Attention and Improved Downsampling Strategy for Remote Sensing Images. Vis. Comput..

[28] Di J., Xi K., Yang Y. (2025). An Enhanced YOLOv8 Model for Accurate Detection of Solid Floating Waste. Sci. Rep..

[29] Li G., Ren G., Wang J., Zhi M., Yu Z., Jiang B., Guan H., Guo Q. (2025). Multimodal fusion transformer network for multispectral pedestrian detection in low-light condition. Sci. Rep..

[30] Sun J., Yin M., Wang Z., Xie T., Bei S. (2024). Multispectral Object Detection Based on Multilevel Feature Fusion and Dual Feature Modulation. Electronics.

[31] Hu J., Shen L., Sun G. Squeeze-and-Excitation Networks. Proceedings of the IEEE Conference on Computer Vision and Pattern Recognition (CVPR).

[32] Howard A.G., Zhu M., Chen B., Kalenichenko D., Wang W., Weyand T., Andreetto M., Adam H. (2017). MobileNets: Efficient Convolutional Neural Networks for Mobile Vision Applications. arXiv.

[33] Wei X., Li Z., Wang Y. (2025). SED-YOLO based multi-scale attention for small object detection in remote sensing. Sci. Rep..

[34] Qu S., Dang C., Chen W., Liu Y. (2025). SMA-YOLO: An improved YOLOv8 algorithm based on parameter-free attention mechanism and multi-scale feature fusion for small object detection in UAV images. Remote Sens..

[35] Wang Q., Yu C. (2025). AMFE-YOLO: A Small Object Detection Model for Drone Images. IET Image Process..

[36] Vaswani A., Shazeer N., Parmar N., Uszkoreit J., Jones L., Gomez A.N., Kaiser L., Polosukhin I. Attention is all you need. Proceedings of the Advances in Neural Information Processing Systems (NeurIPS).

[37] Cui C., Xie J., Yang Y. (2023). Bright channel prior attention for multispectral pedestrian detection. arXiv.

[38] Chen Y., Dai X., Liu M., Chen D., Yuan L., Liu Z. (2019). Dynamic Convolution: Attention over Convolution Kernels. arXiv.

[39] Yang B., Bender G., Le Q.V., Ngiam J. (2019). CondConv: Conditionally Parameterized Convolutions for Efficient Inference. arXiv.

[40] Liu J., Fan X., Huang Z., Wu G., Liu R., Zhong W. Target-aware Dual Adversarial Learning and a Multi-scenario Multi-Modality Benchmark to Fuse Infrared and Visible for Object Detection. Proceedings of the IEEE/CVF Conference on Computer Vision and Pattern Recognition (CVPR).

[41] Razakarivony S., Jurie F. (2016). Vehicle Detection in Aerial Imagery: A Small Target Detection Benchmark. J. Vis. Commun. Image Represent..

[42] Jia X., Zhu C., Li M., Tang W., Zhou W. LLVIP: A Visible-infrared Paired Dataset for Low-light Vision. Proceedings of the 2021 IEEE/CVF International Conference on Computer Vision Workshops (ICCVW).

[43] Zhang J., Lei J., Xie W., Fang Z., Li Y., Du Q. (2023). SuperYOLO: Super Resolution Assisted Object Detection in Multimodal Remote Sensing Imagery. IEEE Trans. Geosci. Remote Sens..

[44] Chen Y., Wang B., Guo X., Zhu W., He J., Liu X., Yuan J., Antonacopoulos A., Chaudhuri S., Chellappa R., Liu C.-L., Bhattacharya S., Pal U. (2025). DEYOLO: Dual-Feature-Enhancement YOLO for Cross-Modality Object Detection. Pattern Recognition. ICPR 2024.

[45] Shen J., Chen Y., Liu Y., Zuo X., Fan H., Yang W. (2023). ICAFusion: Iterative Cross-Attention Guided Feature Fusion for Multispectral Object Detection. Pattern Recognit..

[46] Shao Y., Huang Q., Mei Y., Chu H. (2024). MOD-YOLO: Multispectral Object Detection Based on Transformer Dual-Stream YOLO. Pattern Recognit. Lett..

[47] Yang D., Zhang X., Wang P. (2026). FID-YOLO: A pedestrian detection model integrating multispectral information in complex environments. PLoS ONE.

[48] Sharma M., Dhanaraj M., Karnam S., Chachlakis D.G., Ptucha R., Markopoulos P.P., Saber E. (2021). YOLOrs: Object Detection in Multimodal Remote Sensing Imagery. IEEE J. Sel. Top. Appl. Earth Obs. Remote Sens..

